# Unconventional $\left\{10\bar{1}2\right\}$ twinning assisted by pyramidal II stacking faults

**DOI:** 10.1080/21663831.2024.2406910

**Published:** 2024-10-28

**Authors:** Yang Hu, Dennis M. Kochmann

**Affiliations:** Mechanics & Materials Lab, Department of Mechanical and Process Engineering, ETH Zürich, Zürich, Switzerland

**Keywords:** Twin nucleation, pyramidal slip, magnesium, molecular dynamics

## Abstract

Twinning significantly affects the deformation behavior of hexagonal close-packed Mg, so a thorough understanding of twin nucleation and growth mechanisms is required for enhancing the properties of Mg-based materials. The commonly observed $\left\{10\bar{1}2\right\}$ tension twins have been traditionally linked to 〈c + a〉 dislocation dissociation, which results in zonal dislocations with large Burgers vectors several times that of a single twinning dislocation and some residual dislocations. Contrarily, our molecular dynamics simulations reveal $\left\{10\bar{1}2\right\}$ twin nucleation from pyramidal II stacking faults through atomic shuffling without shear displacements. This introduces an alternative twin nucleation mechanism, different from the classically accepted mechanism of dislocation dissociation.

## IMPACT STATEMENT

Unconventional $\left\{10\bar{1}2\right\}$ twin nucleation from pyramidal II stacking faults via atomic shuffling was observed, contradicting the proposed nucleation from pyramidal dislocation dissociation and providing design solutions for hcp metals.

## GRAPHICAL ABSTRACT

**Figure:**
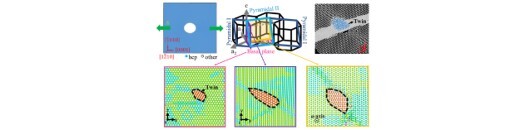

## Introduction

In recent years, magnesium (Mg) has emerged as a promising metal for diverse applications from the automobile and aeronautic industries to the biomedical sector [1,2]. Under external loads, several slip and twin systems can be activated during the deformation of hexagonal close-packed (hcp) Mg and Mg alloys [3–5]. Notably, $\left\{10\bar{1}2\right\}\langle \bar{1}011\rangle$ tension twins were reported to be formed at ∼4 MPa stress in Mg single crystals [6,7]. Besides the easy activation of certain twin types, forming desired twin structures is beneficial for enhancing the strength and ductility of Mg and Mg alloys [8–10]. Therefore, a thorough understanding of the twin nucleation and growth mechanisms is necessary for optimizing twin structures and tuning the overall mechanical properties of Mg-based alloys.

Two major twin nucleation mechanisms reported in literature include (i) nucleation from dislocation dissociation or reaction [11–18], and (ii) nucleation through pure atomic shuffling [19–21]. In 1970s, the nonplanar dissociation of basal, prismatic, and pyramidal dislocations was proposed based on the hcp crystallography, resulting into twinning partials on various twin planes and stair-rod dislocations [11]. Some of the proposed twinning mechanisms were supported by experimental observations [13–15] and atomistic simulations [16,17]. Besides the abovementioned dislocations, zonal dislocations with cores spreading over several twin planes can also dissociate to nucleate twins, as reported in [18]. In contrast to the first category of twinning mechanisms, Wang et al. [19] observed a $\left\{10\bar{1}2\right\}$ twin nucleation at grain boundaries, where the transformation from basal lattices in the parent into prismatic lattices in the twin occurred and involved zero shear. This twin nucleation process was confirmed in nanosized Re crystals during in-situ deformation observations [20]. More recently, atomic shuffling assisted by the migration of partial dislocations on the close-packed planes was shown, leading to the formation of a quasi face-centered cubic (fcc) twinning precursor before the final transformation into the twin [21].

The nucleation of $\left\{10\bar{1}2\right\}$ twins, being the most profuse ones during the plastic deformation of Mg [22], is of great interest and widely discussed. Most studies on $\left\{10\bar{1}2\right\}$ twin nucleation from dislocation dissociation/reaction have revolved around the role of basal slips and associated stacking faults (SFs), as these are the most frequently observed slip systems in Mg [13,15,16]. However, pyramidal dislocations are also crucial in deformed Mg. The activation of such dislocations has been observed in nanocrystalline Mg with grain sizes smaller than 100 nm, which yields at ∼2 GPa [23]. The dissociation of pyramidal 〈c + a〉 dislocations was shown to explain the origin of brittleness of Mg [24], and enhancing the cross-slips of these dislocations can improve ductility [25]. Although mixed 〈c + a〉 dislocations were proposed to dissociate and generate twinning dislocations on $\left\{10\bar{1}2\right\}$ planes [26], this process has not been confirmed experimentally or through simulations.

To address this gap in current understanding, we here report results of MD simulations that mimic the tensile testing of Mg nanopillars. Simulations reveal the activation of partial pyramidal I ( $\left\{10\bar{1}1\right\}$ plane) dislocations and their subsequent cross-slip to the pyramidal II plane ( $\left\{11\bar{2}2\right\}$ plane), followed by the formation of $\left\{10\bar{1}2\right\}$ tension twins from the pyramidal II SF. We demonstrate that such twin nucleation occurs through pure atomic shuffling without the involvement of dislocations, in contrast to the reported twin formation via pyramidal dislocation dissociation.

## Methods

**Figure 1.**
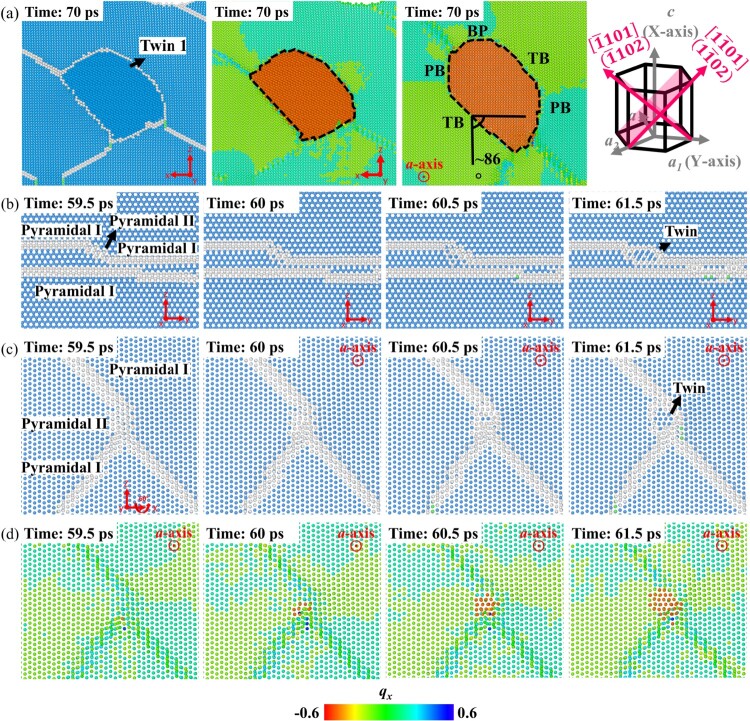
(a) The atomic structure of one $\left\{10\bar{1}2\right\}$ twin activated during the simulation, shown in orange. Atoms are colored according to the component *q_x_* of the orientation quaternion. The corresponding twin variant is shown using the unit cells on the right. To determine the twin type, the simulation box is rotated so that the *Y*-axis after rotation becomes the *a*-axis shared by both twin and matrix. A 1-nm slab containing the twinned region is then used. The twin nucleation process viewed along the (b) *c*-axis and the (c) shared *a*-axis. hcp atoms are blue, fcc atoms are green, while atoms of other types are white. (d) The same view as in (c) with atoms being colored according to *q_x_*.

The MD simulation box before deformation is shown in Figure 1(a). Its *X*-axis is along the [0001]-direction (*c*-axis), the *Y*-axis parallel to the $\left[1\bar{2}10\right]$-direction (*a*-axis), and the *Z*-axis parallel to the $\left[\bar{1}010\right]$-direction of the hexagonal close-packed (hcp) crystal. A nano-sized cylindrical cavity was introduced at the center of the simulation box as the source of defects. Tensile strain increments of 0.5% were applied along the *X*-axis of the box, from 0 up to 5.5% where dislocations were activated. At each strain level, the box was kept at 0.1 K for 30 ps to relax the atomic positions at a fixed temperature and applied strain (i.e. constant strains were maintained for 30 ps at each strain increment). The recorded change of the applied strain, the potential energy, and the normal stress along the *X*-axis with time is shown in Figure S1. Simulations were performed using the Large-scale Atomic/Molecular Massively Parallel Simulator (LAMMPS) package [26] with the modified embedded-atom method (MEAM) potential of Kim et al. [27] to describe the atomic interactions in Mg.

## Results

At 5.5% applied strain, dislocations start to nucleate from the surface of the cylindrical void (Figure S3(a)); such dislocations are determined to be edge-type partial dislocations on pyramidal I planes, with a Burgers vector of $\frac{1}{2}\cdot \frac{1}{2}\langle 10\bar{1}\bar{2}\rangle$, leaving behind a SF on the pyramidal I plane. Detailed analysis of the dislocation type and Burgers vector can be found in Section S2. The SF energy estimated by the MEAM potential is ∼159.04 mJ/m^2^, being consistent with the reported DFT value in [28] (calculations on the SF energy are detailed in Section S3). As the dislocations on differently-oriented pyramidal I planes migrate out of the simulation box, they start to approach and cross each other due to the periodic boundary condition set for all dimensions of the simulation box. The pyramidal I partial dislocations maintain the same characters after penetrating each other (Figure S5), and dislocation activities on different slip planes are also observed, shown as the steps on the initially flat pyramidal I SF (see the middle frame of Figure S3(c)). These dislocations are mixed partial dislocations on pyramidal II planes, with a Burgers vector of $\frac{2-\sqrt{3}}{3}\langle 11\bar{2}3\rangle$, leaving SFs on pyramidal II planes with a thickness of four atomic planes (Figure S3(d)). Figure S7 and Video 3 present the cross-slip of partial dislocations that are first formed on pyramidal I planes to pyramidal II planes and then back to pyramidal I planes that are parallel to previous ones.

Following the cross-slip of pyramidal dislocations, twins start to nucleate, with one example shown in Figure 1(a). According to the component *q_x_* of the orientation quaternion, the twinned region appears in orange color, and the misorientation between the basal planes in the matrix and those in the twin is measured as ∼86°, close to the misorientation across the twin boundary (TB) for $\left\{10\bar{1}2\right\}$ twins. Besides the thus identified $\left\{10\bar{1}2\right\}$ twin plane, basal-prismatic/prismatic-basal (BP/PB) interfaces are part of the boundaries that separate the twin and the matrix. To understand the twinning mechanism, we compare the atomic structures before and after the nucleation of the orange twin. Figure 1(b) shows two pyramidal I SFs (top and bottom) viewed along the *c*-axis, with the top one cross-slipped to the pyramidal II plane, shown by the white atoms (non-hcp atoms) that appear on small, tilted planes. At ∼60 ps, the twin starts to form from the pyramidal II SF and expands rapidly. Figure 1(c) and (d) reveal the same twinning process from the shared *a*-axis between the twin and parent. In those figures, the dislocation that moves from the bottom right corner towards the top left corner is the pyramidal I dislocation that appears at the top of Figure 1(b), while the one that migrates from the bottom left corner and intersects the previous one near the pyramidal II SF is the one that appears at the bottom of Figure 1(b). Importantly, Figure 1(c) and (d) again show that *the twin embryo first emerges at the site of the pyramidal II SF, and pyramidal II planes are non-invariant planes for* $\left\{10\bar{1}2\right\}$ *twins*. Such twins formed from a non-invariant plane under slight distortion have been classified as ‘*unconventional twins*' in recent works [29–31]. A rapid growth follows the twin nucleation, and the major twin facets include TBs, BP/PBs, and twist pyramidal-pyramidal (Twist-PyPy1) and tilt pyramidal-pyramidal (Tilt-PyPy1) facets (the $\left\{10\bar{1}1\right\}$ planes in the matrix facing the $\left\{10\bar{1}1\right\}$ planes at different orientations in the twin), see Figure S8. Such planes were indeed observed as twin interfaces experimentally, using high resolution transmission electron microscopy [32,33].

In addition to the twin shown in Figure 1, another $\left\{10\bar{1}2\right\}$ twin with a different twin variant was formed on a differently-oriented pyramidal II SF (Figure S9). Though this twin is unstable and eventually disappears, this shows the possibility of activating different twin variants by activating pyramidal II SFs of different orientations.

**Figure 2.**
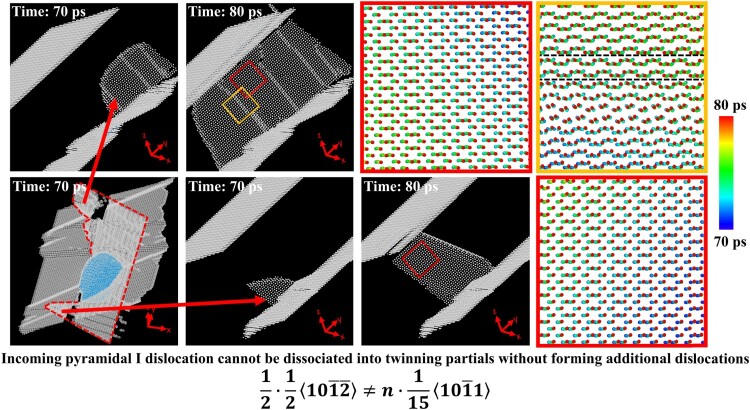
Determining the dislocation types after twin nucleation. hcp atoms are blue, while atoms of other types are white. The incoming pyramidal I dislocation is marked by red dotted lines in the leftmost bottom frame. The positions of atoms in regions outlined by the solid yellow or red rectangles are tracked from 70 to 80 ps, and the atomic displacements are shown in the frames on the right (boxed in the respective colors).

Since an incoming pyramidal I dislocation intersects the pyramidal II SF from which twin nucleates, the question arises if any dislocation dissociation or reaction is involved in the nucleation process. As reported in [12], full pyramidal dislocations (of Burgers vector $\frac{1}{3}\langle 11\bar{2}3\rangle$) can dissociate into zonal twinning dislocations, which nucleate a twin embryo. Yet, in our simulations the activated pyramidal dislocations are partial dislocations with smaller Burgers vectors. Moreover, in most reports on twin nucleation via dislocation dissociation or reaction [11,12], residual dislocations were formed additional to zonal twinning dislocations. We observe that, after twin nucleation, the incoming pyramidal I dislocation is stuck at the SF for some time but eventually penetrates it at certain locations (Figure 2). The dislocations formed after the penetration are still partial pyramidal I dislocations, which cross-slip to the pyramidal II plane and then back to the pyramidal I plane. No new types of dislocations are found. Therefore, *twins in this scenario are more likely formed via pure atomic shuffling*. The contribution of the incoming dislocation is to provide a stress concentration that allows for twin nucleation, shown in Figure S10, which also proves the necessity of pyramidal II SFs in twin nucleation. Even though there is a larger area of stress concentration near the surface of the cylindrical void, twins are not nucleated there.

**Figure 3.**
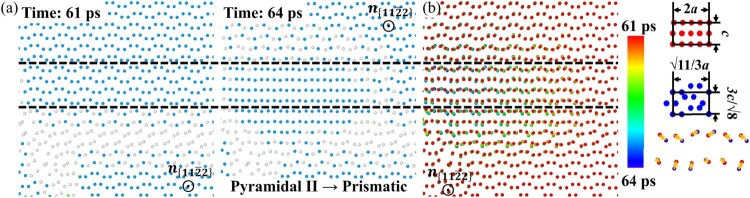
(a) The atomic structure of the twin and its surroundings before (61 ps) and after (64 ps) nucleation. A 0.8-nm slab that is ∼7 Å above the pyramidal II SF is taken. hcp atoms are blue, while atoms of other types are white. (b) Atomic positions during the twin transformation. Atoms are colored to indicate the simulation time, with dark blue for 61 ps and red for 64 ps. Black dashed lines highlight atoms partaking in twinning. The inset in (b) shows the lateral size of the pyramidal II and the prismatic lattice as well as the atomic displacements from 61 ps to 64 ps.

The atomic displacements during the twin transformation are revealed in Figure 3, showing that the pyramidal II planes in the matrix transform into the prismatic planes in the twin, and no shear displacements of atoms are observed. The two directions parallel to the pyramidal II plane, i.e. the $\left\langle 1\bar{1}00\right\rangle$- and $\left\langle 11\bar{2}3\right\rangle$-directions, become the ⟨0001⟩- and $\left\langle 1\bar{2}10\right\rangle$-directions parallel to the prismatic plane, respectively. The pyramidal II lattice is indeed close to the prismatic lattice. The repeating units of the two lattices are shown in the inset of Figure 3(b). The length and width of the repeating unit of the prismatic lattice are 2*a* and *c*, respectively (where *a* and *c* are the lattice parameters of Mg), compared to, respectively, $\sqrt{11/3}$*a* and $3/\sqrt{8}$*c* for the pyramidal II lattice. The distance between two prismatic planes along the plane normal is about $\sqrt{3}/2$*a*, which is close to 0.853*a* for the pyramidal II lattice (Figure S11). The similar sizes of the pyramidal II and prismatic lattices likely imply a low energy barrier for the twin transformation.

Analogous simulations were conducted at room temperature, and twin nucleation from pyramidal II SFs was again observed (Section S9), meaning that the twinning mechanism proposed here is not limited to low temperatures (while the low-temperature results are cleaner to interpret). At higher temperatures, stresses/strains required for twin nucleation via atomic shuffling become lower, indicating that temperature can reduce the energy barrier for atoms to move from their initial positions in the matrix to the final positions in the twin (Figure S13). While the contribution of pyramidal II SFs is more profound at low simulation temperatures.

**Figure 4.**
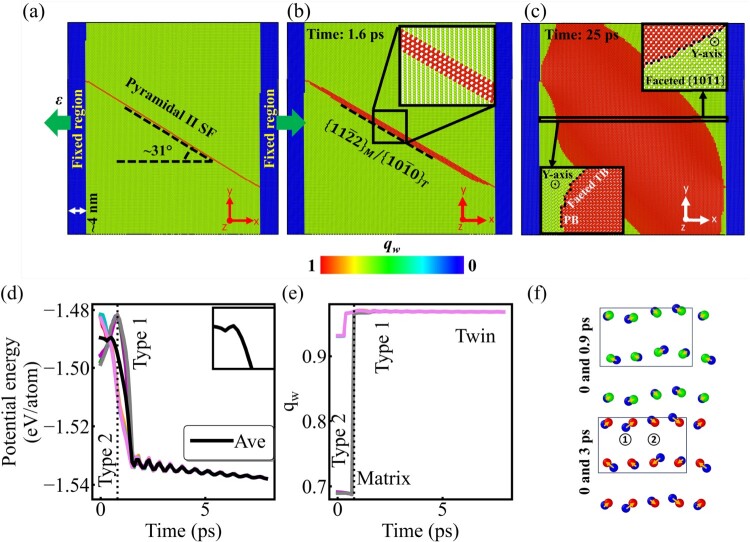
(a) The initial configuration of the simulation box containing one pyramidal II SF. The twin structure at 5.5% tensile strain (b) and at 1.6 ps, (c) at 25 ps. Insets show zoomed-in views of various twin facets. Atoms are colored by the lattice orientation component *q_w_*. The variation of (d) atomic PE and (e) *q_w_* with time for eight atoms shown in (f). The inset in (d) shows a zoomed-in view of the average PE/atom from −1.545 to −1.478 eV. (f) The atomic displacements from 0 to 0.9 ps, and from 0 to 3 ps.

The twin nucleation from pyramidal II SFs via atomic shuffling is further confirmed by additional simulations with a single pyramidal II SF in the simulation box (Section S10). The same protocol of applying tensile strain increments as in previous simulations was used to activate twin nucleation, which starts from the SF as expected. Subsequently, the twin propagates rapidly along the pyramidal II plane, adopting a laminate shape (Figure 4). Thickening of the twin follows; again the $\left\{11\bar{2}2\right\}$/ $\left\{10\bar{1}0\right\}$ interfaces disappear, and the major twin facets eventually become faceted TBs, BP/PBs Twist-PyPy1 and Tilt-PyPy1 facets. The atomic displacements at the nucleation stage, similar to what is shown in Figure 3, differ strongly from those during TB migration, a process that requires both shear and shuffle displacements, indicating the existence of interface dislocations (Figure S17). Eight atoms are the minimum unit necessary to render the atomic arrangements for the observed twin nucleation (see inset in Figure 4), which can be grouped into two types. One type undergoes small shuffling displacements and releases energy during twin transformation. The other type undergoes larger shuffling displacements and must overcome a ∼15.12 meV potential energy (PE) barrier. The top frame in Figure 4(f) shows the intermediate state of type 2 atoms during their motion towards their positions in the twin. However, when the PE of the whole unit is tracked with time, the PE barrier for twin nucleation becomes negligible, implying that the energy barriers required to be overcome by type 2 atoms are compensated by the easy-move type 1 atoms, and the whole unit requires little energy to be transformed into the twin. This is evidence of defect-assisted twin nucleation requiring significantly less energy. The Gibbs free energy barrier was also estimated and is presented in Section S11, showing similar trends as the PE barriers.

## Discussion

Our study, while focused on Mg, has broader implications for other hcp metals, especially those with a similar c/a ratio to Mg. The alternative $\left\{10\bar{1}2\right\}$ twin nucleation mechanism we observed, involving pyramidal II SFs and atomic shuffling, could be applicable to a wide range of hcp metals and alloys, offering a new perspective on their deformation behavior. Our findings can also contribute to understanding the formation of ‘unconventional twins' or ‘axial weak twins', which are emerging concepts to describe twins formed on non-invariant planes, and such planes do not contain the necessary twinning partials for twin nucleation [29–31].

The simulated twin nucleation occurs at ∼4.3 GPa (the estimated tensile stress at the core of the incoming pyramidal I dislocation), which is considerably higher than the nucleation stress of $\left\{10\bar{1}2\right\}$ twins in polycrystalline Mg with grain sizes in the micro-meter scale [6,7]. Yet, such high stresses to activate twin or dislocation nucleation have indeed been reported for nanocrystalline metals. For example, in [20], a $\left\{10\bar{1}2\right\}$ twin nucleation in the compression region near the core of a matrix dislocation was shown, with the stress being estimated at ∼5 GPa. The onset of pyramidal I dislocations in our simulations is at ∼2.96 GPa, being comparable to the ∼2 GPa yield strength of Mg nano-polycrystals reported in [23], in which the motion of pyramidal dislocations was observed in-situ, in addition to easily-activated basal slips.

As grain size decreases in the coarse-grained regime, the tendency for materials to twin reduces [34,35]. Meyers et al. [36] reported a Hall-Petch-type relationship, showing that the critical resolved shear stress (CRSS) for twinning often exhibits a stronger dependence on grain size than the CRSS for slip. This suggests that the CRSS for $\left\{10\bar{1}2\right\}$ twinning may increase and potentially approach that of pyramidal dislocations as grain size decreases. In the nano-grained regime, twinning is rarely observed [37] and the grain size effect on twinning is uncertain [38]. Our simulations reveal a possibility to nucleate $\left\{10\bar{1}2\right\}$ twins with the assistance of pyramidal II SFs, providing an opportunity to tune the mechanical properties of nanocrystalline hcp metals and alloys. The dissociation of pyramidal dislocations and SF energies can be tailored through alloying [39]. The addition of solute elements can also promote the onset of pyramidal II dislocations or enhance cross-slips of pyramidal I dislocations [25], pointing out pathways for alloy design.

Moreover, for heavily deformed materials, such as those processed by equal channel angular extrusion, the activation of easy slip systems like basal slips can saturate after certain strains due to strain hardening, changes in the lattice orientation due to twinning, and grain refinement [40]. Under these conditions, further twinning through dissociation/reaction of easily-activated dislocations and associated SFs may become limited. Our proposed mechanism offers an alternative route for twinning, which could be particularly beneficial in such strain-hardened materials.

This mechanism also sheds light on understanding the deformation of materials under extreme conditions, such as impact loading, where rapid stress increases can occur. Under impact loading, the activation of pyramidal dislocations, alongside other slip and twin systems, could be significant [41,42]. Our study provides a foundational understanding that could guide the development of Mg and other hcp metals and alloys to withstand such extreme conditions, enhancing their performance in applications ranging from automotive to aerospace industries.

## Conclusion

In summary, our MD simulations reveal the possibility of $\left\{10\bar{1}2\right\}$ twin nucleation assisted by the SF on a pyramidal II plane, a non-invariant plane of $\left\{10\bar{1}2\right\}$ twins. Such twin nucleation cannot be explained by the common assumption of the dissociation of pyramidal 〈c + a〉 dislocations into twinning dislocations on the twin plane. Instead, it occurs through pure atomic shuffling. Our findings propose a twin nucleation mechanism at high stresses, being relevant to various circumstances such as the deformation of nanocrystalline hcp metals and alloys, metals and alloys under severe plastic deformation, and deformation under impact loading. Our work not only contributes to the fundamental understanding of twin nucleation mechanisms, but also offers practical insights for alloy design, particularly in fine-tuning the mechanical properties of hcp metals and alloys under varying conditions. This broader applicability underscores the general impact of our findings. Our work also calls for a more detailed, quantitative comparisons of the energy barrier for twin transformation through dislocation dissociation versus the twin transformation via atomic shuffling and assisted by pyramidal II SFs.

## Supplementary Material

Supplemental Material

Hu_MRL_SM_DMK.docx
