# Supplementary figures and images for: Unconventional {101¯2} twinning assisted by pyramidal II stacking faults
Source: Mater Res Lett. 2024 Oct 28;13(1):1–8. doi: 10.1080/21663831.2024.2406910 (PMC11737610; doi:10.1080/21663831.2024.2406910)

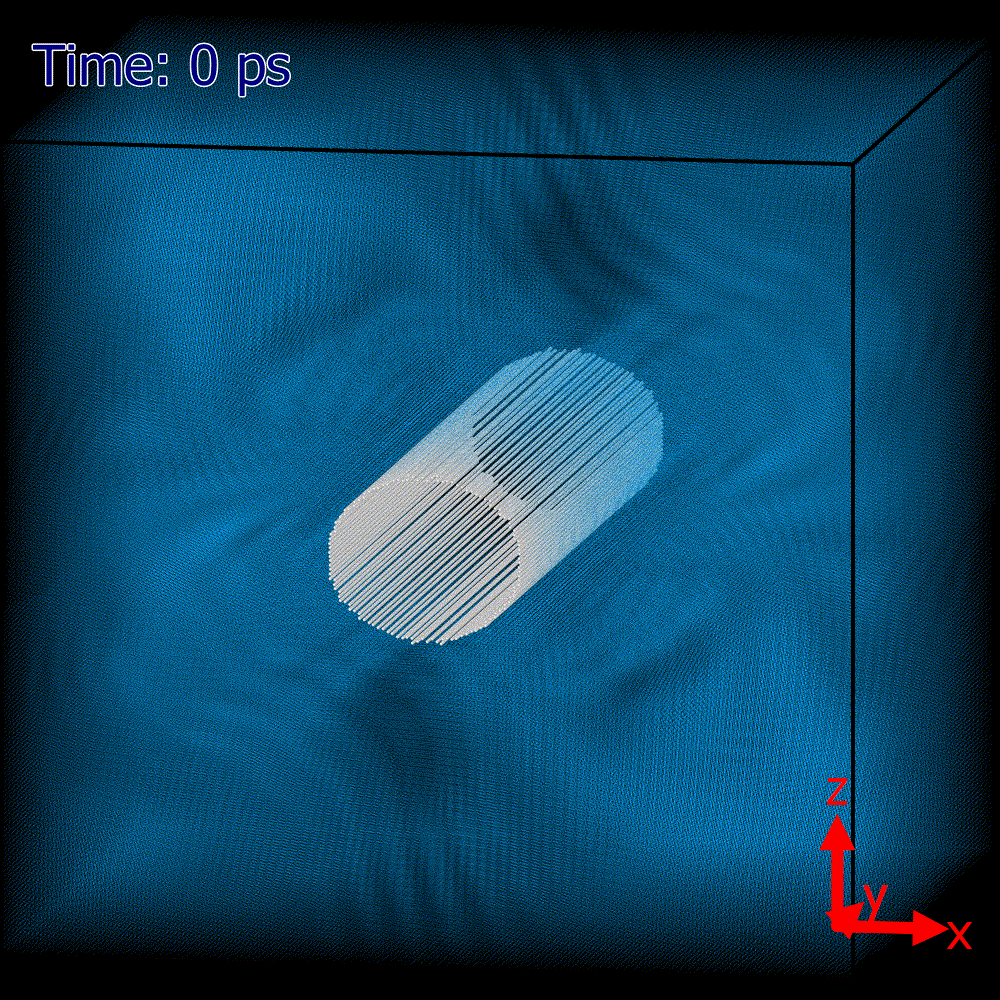

Supplement: Supplemental Material [file TMRL_A_2406910_SM2499.zip › Supplementary videos/Video1_slip.gif]

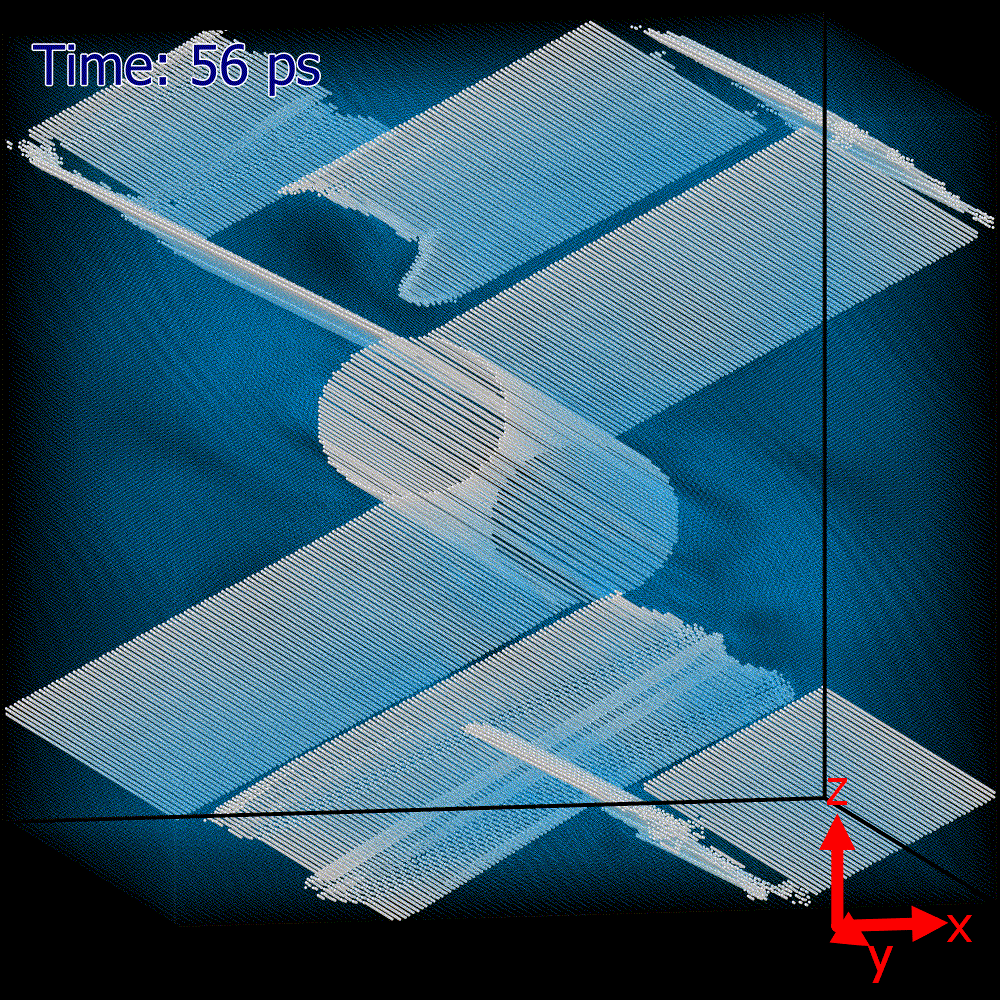

Supplement: Supplemental Material [file TMRL_A_2406910_SM2499.zip › Supplementary videos/Video2_twin.gif]

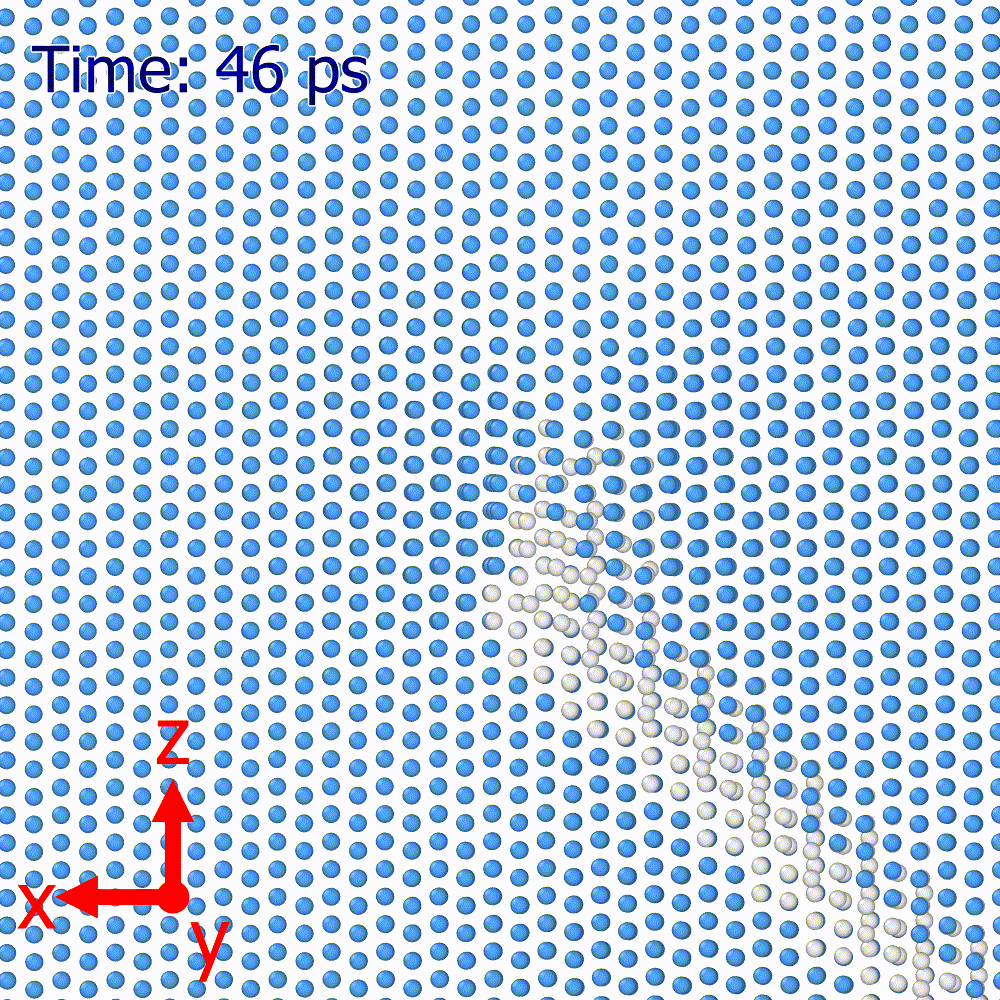

Supplement: Supplemental Material [file TMRL_A_2406910_SM2499.zip › Supplementary videos/Video3_cross_slip.gif]

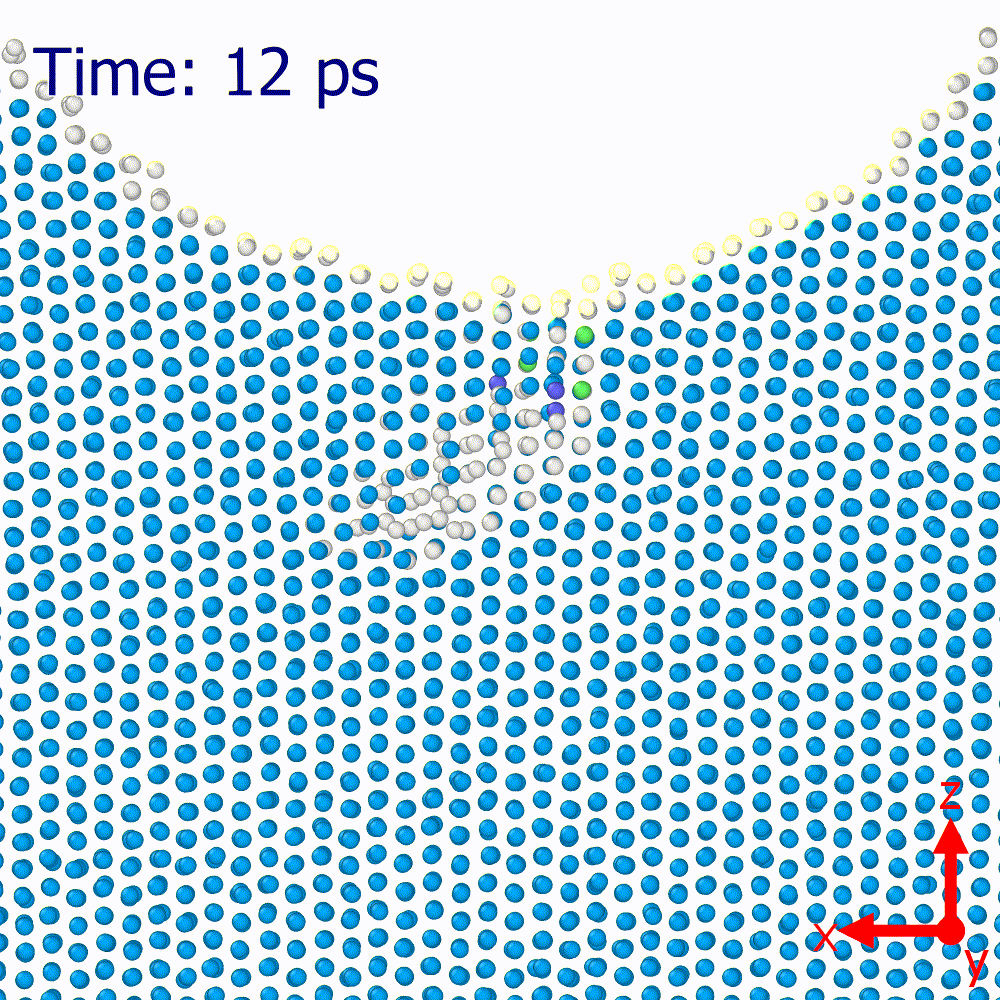

Supplement: Supplemental Material [file TMRL_A_2406910_SM2499.zip › Supplementary videos/Video4_twin_300k.gif]

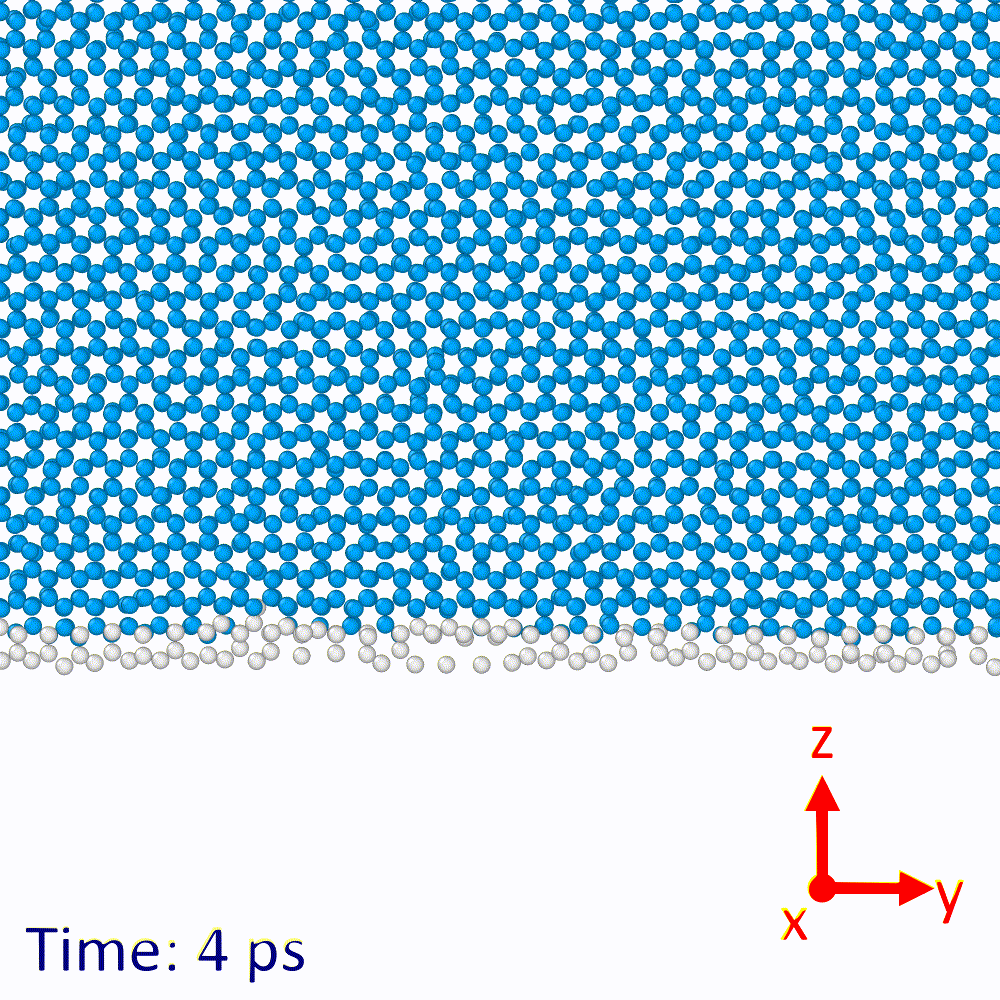

Supplement: Supplemental Material [file TMRL_A_2406910_SM2499.zip › Supplementary videos/Video5_twin_300k_another.gif]
