# Supplementary material for: Unconventional {101¯2} twinning assisted by pyramidal II stacking faults
Source: Mater Res Lett. 2024 Oct 28;13(1):1–8. doi: 10.1080/21663831.2024.2406910 (PMC11737610; doi:10.1080/21663831.2024.2406910)
Supplement: Hu_MRL_SM_DMK.docx [file TMRL_A_2406910_SM0629.docx]

**Unconventional** $\left\{ \boldsymbol{10}\bar{\boldsymbol{1}}\boldsymbol{2} \right\}$ **twinning assisted by pyramidal II stacking faults**

Yang Hu^a^, Dennis M. Kochmann^a, *^

^a^ Mechanics & Materials Lab, Department of Mechanical and Process Engineering, ETH Zürich, 8092 Zürich, Switzerland

* Corresponding author: dmk@ethz.ch

**Supplementary Materials**

**S1. Computational details**

The dimensions of the simulation box are ~50×~50×~50 nm^3^, containing ~5,600,000 atoms. The *XY*-plane is the prismatic plane, i.e., the $\left\{ 10\bar{1}0 \right\}$ plane, the *XZ*-plane is the $\left\{ 1\bar{2}10 \right\}$ plane, and the *YZ*-plane is the basal plane of the hcp lattice, with the atomic structures of each plane shown below. Figure S1(a) presents the applied strain as a function of time. For each 30 ps, a constant strain along the *X*-axis was maintained, while the stresses along the *Y*- and *Z*-axis were relaxed, using an isothermal-isobaric (NPT) ensemble. The change of the potential energy and the normal stress along the *X*-axis with time are shown in Figure S1(b). As the applied tensile strain increases, the stress also increases, as does the potential energy of the box. Before 0.3 ns, since there are no defects being generated in the simulation box, the stress and potential energy reach their plateau values quickly after the strain increments are applied. At 0.3 ns, the total applied tensile strain reaches 5.5%. At this strain level, there is a sudden decrease in the normal stress and the potential energy, indicating the emergence of dislocation activities, see Videos 1 and 2 for the slip and twin activities, respectively, during the deformation of Mg. Simulations at 5% strain were prolonged to 2 ns to ensure that no defects were activated at this strain level, confirming that 30 ps is sufficient for the structural relaxation to the energetically most favorable state at the applied strain and temperature.

Structural visualization was performed using the Open Visualization Tool (OVITO) [1], and the Polyhedral Template Matching method [2] was used to characterize the local crystalline structure and orientation associated with each atom in the system, which can be encoded as an orientation quaternion, ***q*** *= q_w_ + q_x_****i*** *+ q_y_****j*** *+ q_z_****k***. Components *q_x_* and *q_w_* are suitable indicators for twin structures generated during the simulation.


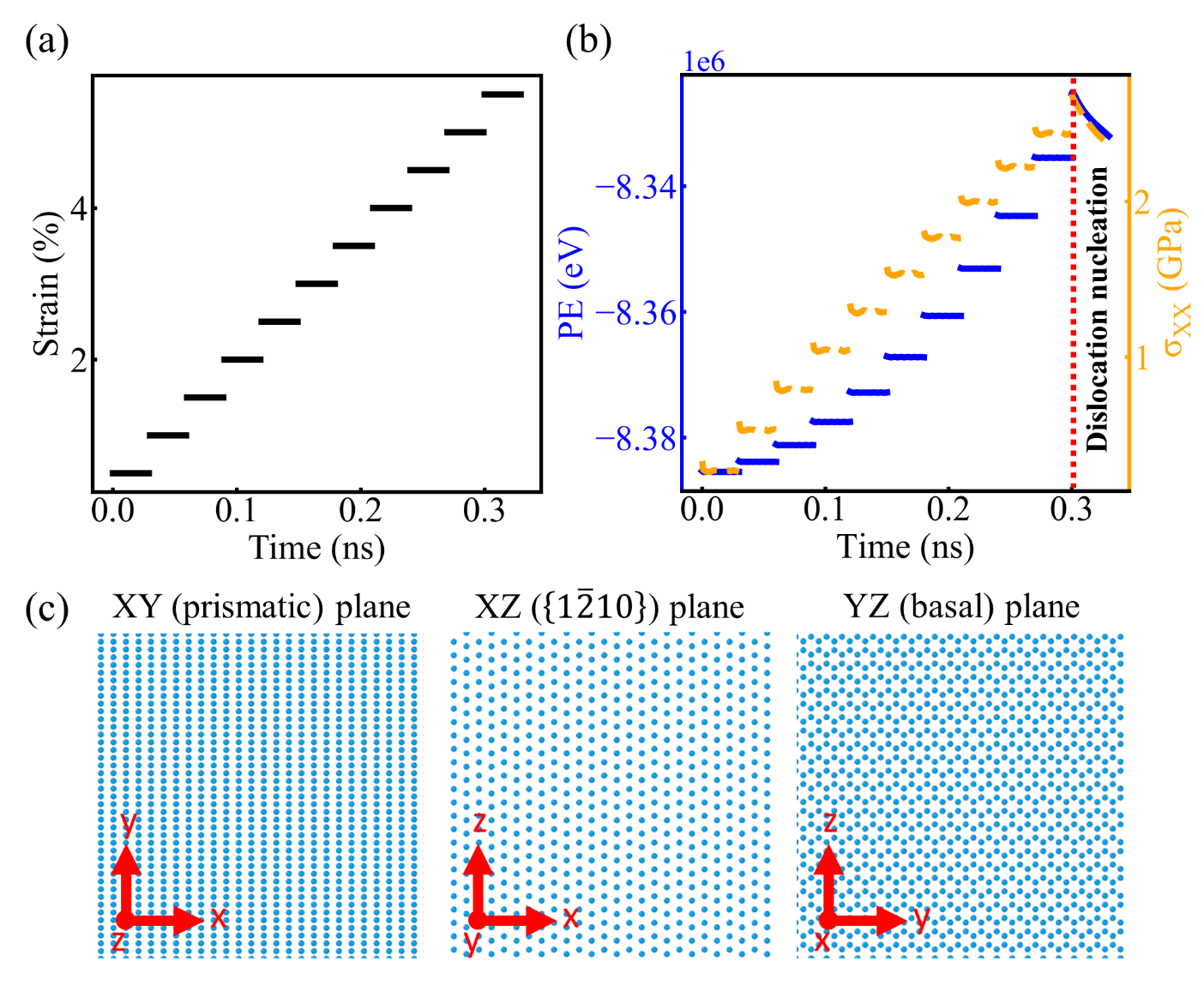


Figure S1. The time evolution of (a) the applied strain, (b) the potential energy and the normal stress along the *X*-axis. (c) The atomic structure of the *XY*-, *XZ*-, *YZ*-planes.


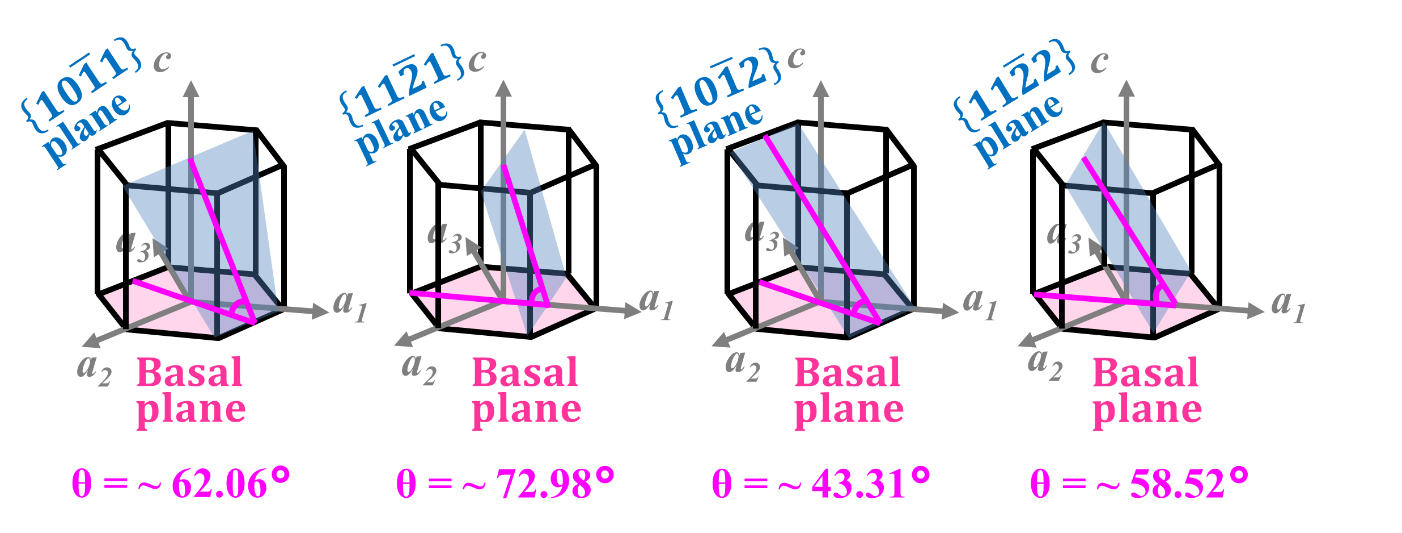


Figure S2. The unit cell of hcp metals, showing the angle between the $\left\{ 10\bar{1}1 \right\}$, $\left\{ 11\bar{2}1 \right\}$, $\left\{ 10\bar{1}2 \right\}$, $\left\{ 11\bar{2}2 \right\}$ plane and the basal plane.

**S2. Dislocations nucleated during the simulation**

For dislocations nucleated from the surface of the cylindrical void (Figure S3(a)), the angle from the slip planes to basal planes about the *X*-axis is ~63°-64°, which is close to the angle between the pyramidal I plane and the basal plane (calculated based on the crystallography of hcp Mg), indicating pyramidal I slip (see Figure S2 for the angles between different crystal planes and basal planes about the *a*-axis). Top views of the slip plane during dislocation motion are presented in the last frame of Figure S3(b), with the time evolution of atomic positions being tracked. The Burgers vector of the dislocation was determined as $\frac{1}{2}\cdot\frac{1}{2}\left\langle10\bar{1}\bar{2} \right\rangle$, which is a partial dislocation, leaving behind a SF on the pyramidal I plane. The dislocations emitted from the void surface are edge dislocations since their dislocation lines are perpendicular to the direction of motion (Figure S4(a)).

As the dislocations on differently-orientated pyramidal I planes migrate out of the simulation box, they start to approach and cross each other due to the periodic boundary condition set for all dimensions of the simulation box. The pyramidal I partial dislocations maintain the same characters after penetrating each other (Figure S5), and dislocation activities on different slip planes are also observed, shown as the steps on the initially flat pyramidal I SF (see the middle frame of Figure S3(c)). The angle between the slip plane and a $\left\{ 1\bar{2}10 \right\}$ plane (a differently-oriented one than the original *XZ*-plane) about the $\left\langle\bar{1}010 \right\rangle$-direction as ~31°, being close to the angle between the $\left\{ 1\bar{2}10 \right\}$ plane and the pyramidal II plane. Hence, such SFs are formed on pyramidal II planes with a thickness of four atomic planes. The Burgers vector is determined as $\frac{2-\sqrt{3}}{3}\left\langle11\bar{2}3 \right\rangle$ using the top views of pyramidal II planes in Figure S3(d). The dislocations formed after the intersection of the pyramidal I dislocations emitted from the void surface are mixed-type, since their dislocation lines are neither perpendicular nor parallel to the direction of motion (Figures S4(b) and S4(c)).


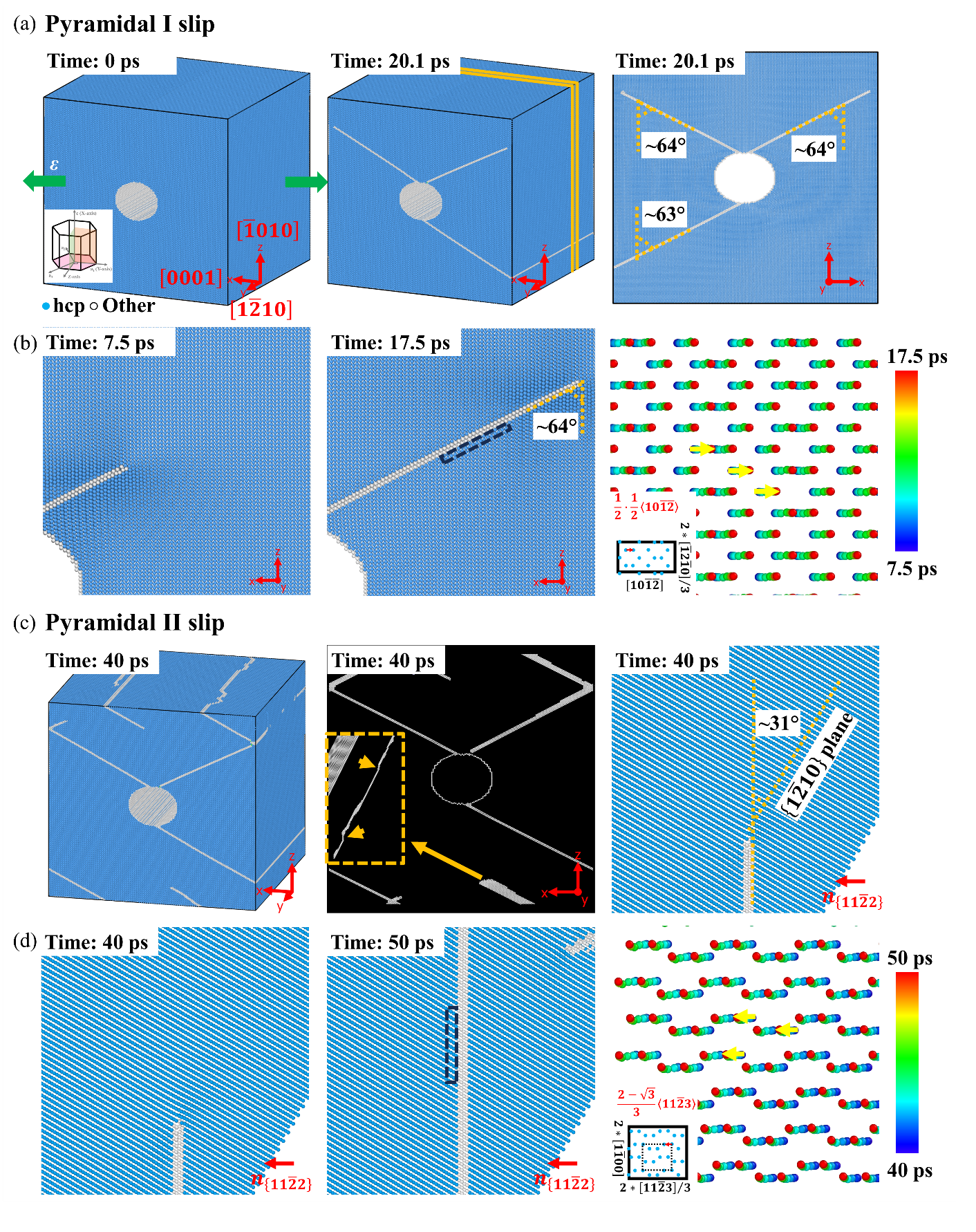


Figure S3. Activation of pyramidal I and II slips during deformation: (a) Slip planes and (b) Burgers vectors for pyramidal I, and (c) slip planes and (d) Burgers vectors for pyramidal II are identified. The first frame in (a) shows the MD setup and the simulation box orientation. hcp atoms are blue, while atoms of other types are white. The final frames of (b) and (d) provide top views of pyramidal I and II planes with atomic trajectories. Atoms are colored by simulation time, transitioning from dark blue (early times) to red (later times). Insets detail the calculated Burgers vectors with red arrows. A two-atomic-plane slab below the pyramidal I slip plane and a four-atomic-plane slab above the pyramidal II slip plane are then extracted for structure visualization (marked using the black box in dashed line). In (c), the dashed yellow box show a side view of the pyramidal II dislocations, with the viewing direction along the $\left\langle11\bar{2}3 \right\rangle$-direction. For the first two frames in (d), the viewing direction is parallel to the pyramidal II slip plane.


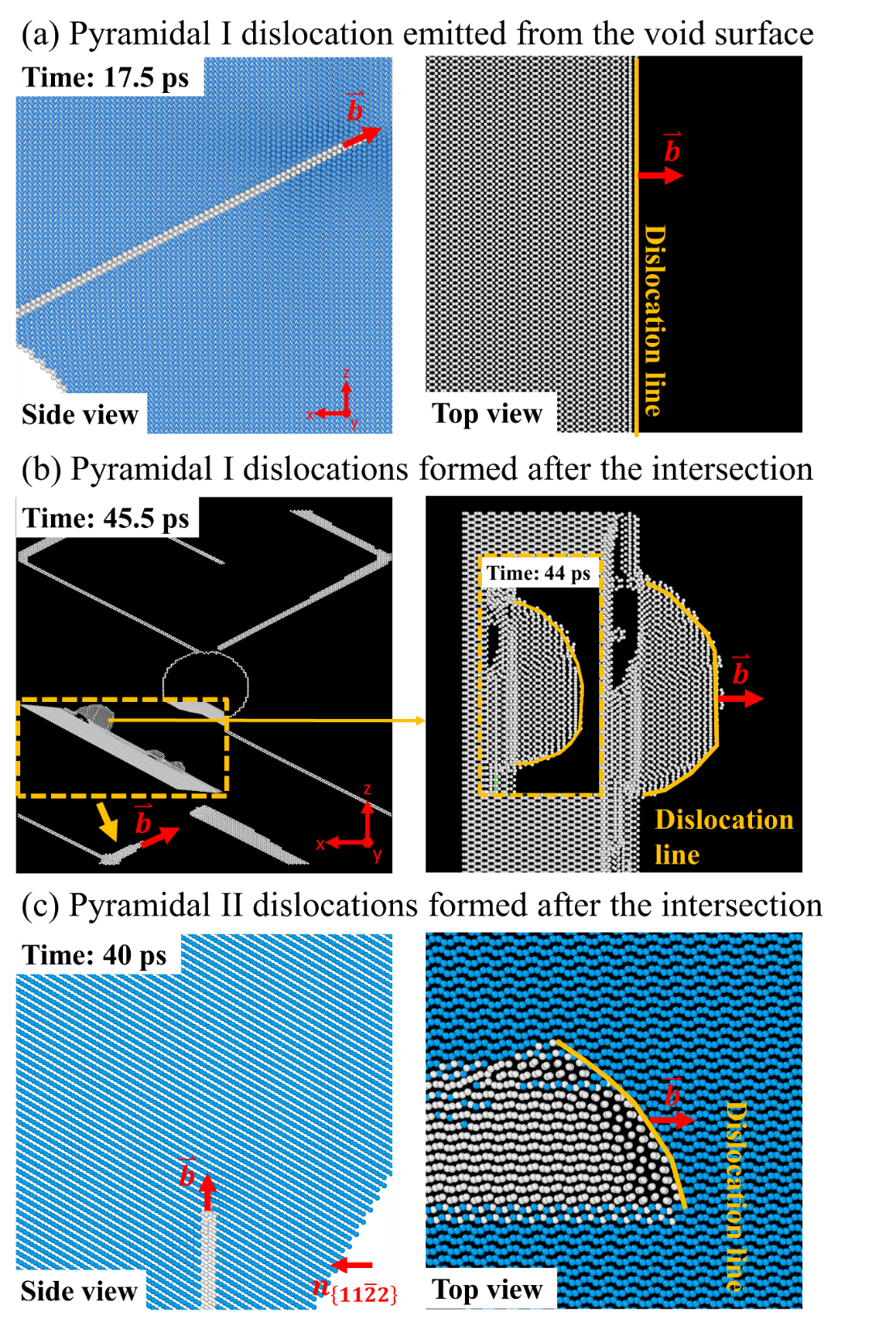


Figure S4. (a) The side view and top view of a partial pyramidal I dislocation emitted from the void surface. This is the same dislocation shown in the middle frame in Figure S3(b). (b) The side view and top view of a partial pyramidal I dislocation formed after the intersection of two differently oriented pyramidal I dislocations. (c) The side view and top view of a partial pyramidal II dislocation. This is the same dislocation shown in the first frame in Figure S3(d).


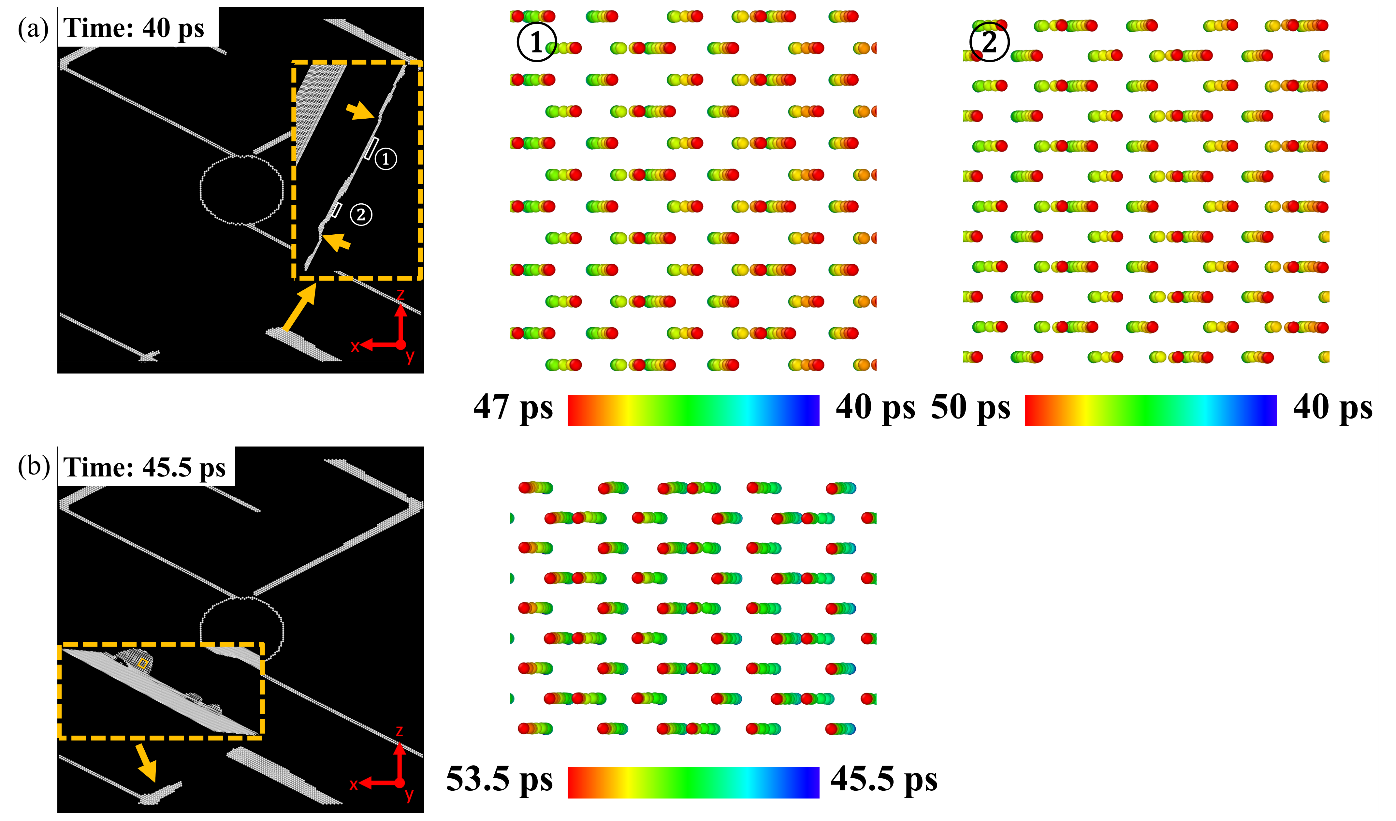


Figure S5. Determining the dislocation types after one pyramidal I dislocation crosses another pyramidal I dislocation migrating on a differently oriented plane, due to the periodic boundary condition. The time evolution of positions of atoms in regions outlined by the solid yellow rectangles are tracked, and the atomic displacements are shown in the frames on the right (boxed in the respective colors). In (a), two regions right below the pyramidal I SF, which is the same one as shown on the top in Figure 1(b), are picked. This is the pyramidal I dislocation which cross-slipped to pyramidal II plane, followed by twin nucleation. In (b), one region right below the pyramidal I SF, which is the same one as shown at the bottom in Figure 1(b), is picked. This is the incoming pyramidal I dislocation which intersects the pyramidal II SF, where the twin nucleates.

**S3. Calculating the stacking fault energy and the interfacial energy**

For calculating the energies of pyramidal stacking faults, simulation boxes containing one stacking fault each were constructed. The simulation box has the same orientation as in Figure S3, while the box dimensions are ~45.2 × 14.4 × 45.5 nm^3^ for the pyramidal I stacking fault and ~45.2 × 45.4 × 15.0 nm^3^ for the pyramidal II stacking fault. To create the same pyramidal stacking fault as observed in previous simulations, half box of atoms are displaced according to the obtained Burgers vector, which is $\frac{1}{2}\cdot\frac{1}{2}\left\langle10\bar{1}\bar{2} \right\rangle$ along the pyramidal I plane and $\frac{2-\sqrt{3}}{3}\left\langle11\bar{2}3 \right\rangle$ along the pyramidal II plane. Some atoms at the edge of the simulation box which became too close to each other were deleted. The periodic boundary conditions were kept for all box dimensions. The simulation box was structurally relaxed at 0 K (molecular statics) with the box volume being allowed to change. After certain steps of energy minimization, the pyramidal stacking fault with the same atomic arrangement as in previous simulations was observed, along with a basal stacking fault at the edge of the box connected to the pyramidal stacking fault. The potential energies of atoms that are far from the stacking fault return to the level for atoms in a bulk sample. Atoms within a region at the center of the box were chosen for calculating the stacking fault energy (marked by the dotted regions in Figure S6). For calculating the interfacial energies, simulation boxes containing two interfaces were constructed, and periodic boundary conditions along all axes were maintained. The distance between two interfaces is over 60 nm to avoid the interaction between them. Among all facets, only the TB is fully relaxed, meaning that the atoms in the bulk regions of the upper and lower parts have the same potential energies as those in a bulk sample. By contrast, coherent basal-prismatic/prismatic-basal (BP/PBs), coherent twist pyramidal-pyramidal (Twist-pypy1) or tilt pyramidal-pyramidal (Tilt-pypy1) and the $\left\{ 11\bar{2}2 \right\}_{M}$/$\left\{ 10\bar{1}0 \right\}_{T}$ interfaces only exist if the two lattices above and below are strained. Introducing misfit dislocations can further relax the stress but also increase the interfacial energies, see Ref. [3,4]. The simulation box containing two $\left\{ 11\bar{2}2 \right\}_{M}$/$\left\{ 10\bar{1}0 \right\}_{T}$ interfaces were obtained from a simulation on twin nucleation from a single pyramidal II stacking fault, and the structure was further relaxed until the interface was about to transform. The interfacial energy was computed as

$\gamma=\left( E_{int/SF}-{n\cdot E}_{atom, bulk} \right)/A$ (1)

where *n* is the total number of atoms in the simulation, *A* is the interfacial area, $E_{int/SF}$ is the potential energy of the structure containing interfaces or stacking faults, while $E_{atom,bulk}$ is the potential energy of an atom in the bulk region. For fully relaxed interfaces, this value is the same as the value calculated using a bulk sample without defects. Yet, for non-relaxed interfaces, the lattices on the two sides of the interface are strained, so that the atomic potential energy used is different from the value obtained from a fully relaxed bulk sample. The energy of the pyramidal I stacking fault estimated by the MEAM potential used in our work is ~159.04 mJ/m^2^, which is close to the reported DFT value in [5]. The Burgers vector of the pyramidal II dislocation observed in our work is smaller than that reported in [5,6]; an estimation of the stacking fault energy using the MEAM potential is ~204.66 mJ/m^2^. In Refs. [5,6], DFT computations showed a pyramidal II stacking fault with a Burgers vector of $\frac{1}{6}\left\langle11\bar{2}3 \right\rangle$, and the reported stacking fault energies calculated using the standard and the full relaxation method were 165 mJ/m^2^ and 236 mJ/m^2^, respectively. Our calculations show that coherent Twist-PyPy1 or Tilt-PyPy1 facets have the lowest energy, viz. 106.69 mJ/m^2^, followed by the coherent BP/PB interface with an energy of 114.17 mJ/m^2^. The energy of a coherent TB is about 143.87 mJ/m^2^, and the $\left\{ 11\bar{2}2 \right\}_{M}$/$\left\{ 10\bar{1}0 \right\}_{T}$ interfaces have the highest interfacial energy of 431.86 mJ/m^2^. These calculated results of coherent TB, BP/PB, coherent Twist-PyPy1, and Tilt-PyPy1 are close to the values reported in the work of Gong et al. [7].


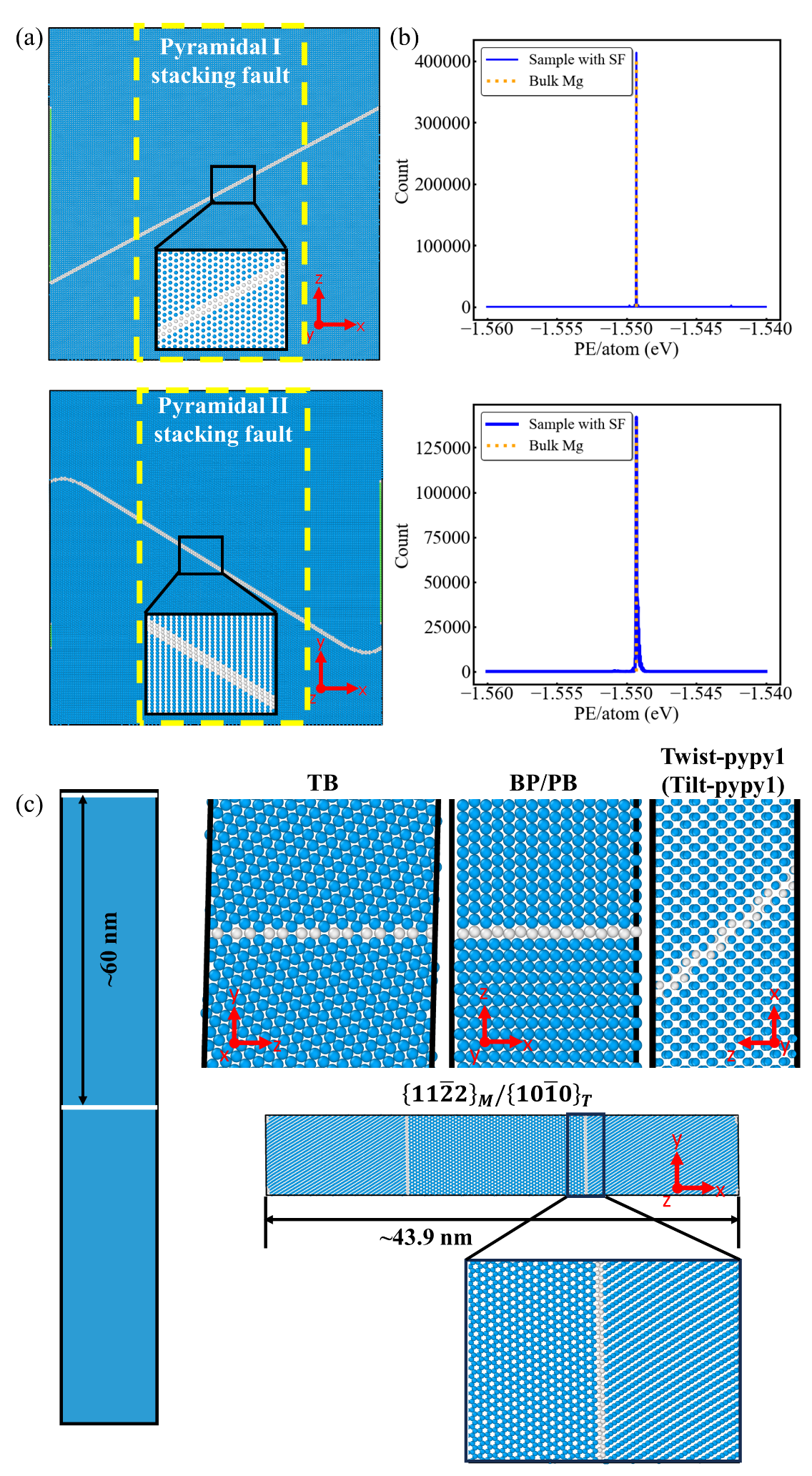


Figure S6. The setup of simulations for calculating stacking fault energies and interfacial energies. The atomic structures of pyramidal I and pyramidal II stacking faults, coherent TBs, coherent BP/PBs, coherent Twist-pypy1 (Tilt-pypy1) and coherent $\left\{ 11\bar{2}2 \right\}_{M}/\left\{ 10\bar{1}0 \right\}_{T}$ planes are shown.

**S4. The cross-slip of dislocations from the pyramidal I plane to the pyramidal II plane**

Figure S7(a) shows the stacking fault structure along a $\left[ 1\bar{2}10 \right]$-direction (the simulation box has been rotated about the *X*-axis by 60°), and only atoms of non-hcp type are displayed. The partial dislocation propagates rightward, and the green arrow marks where the cross-slip occurs. In Figure S7(b) the same stacking fault is viewed along the $\left[ 1\bar{2}10 \right]$-direction with surrounding hcp atoms, after rotating the simulation box about the *X*-axis by -60° (a 1-nm slab is taken for this view). At 45 ps, the white atoms (non-hcp atoms) show the pyramidal I stacking fault before cross-slip and the dislocation moves along a diagonal direction from this perspective. Within the following 1 ps, the dislocation changes its direction of motion to the vertical direction, and this causes the stacking fault on pyramidal II planes. At 48 ps, the partial dislocation returns to its original direction of motion, meaning that it returns to the pyramidal I plane, and the atomic snapshot at 50 ps shows the pyramidal I stacking fault sandwiched by a small area of pyramidal II stacking fault.


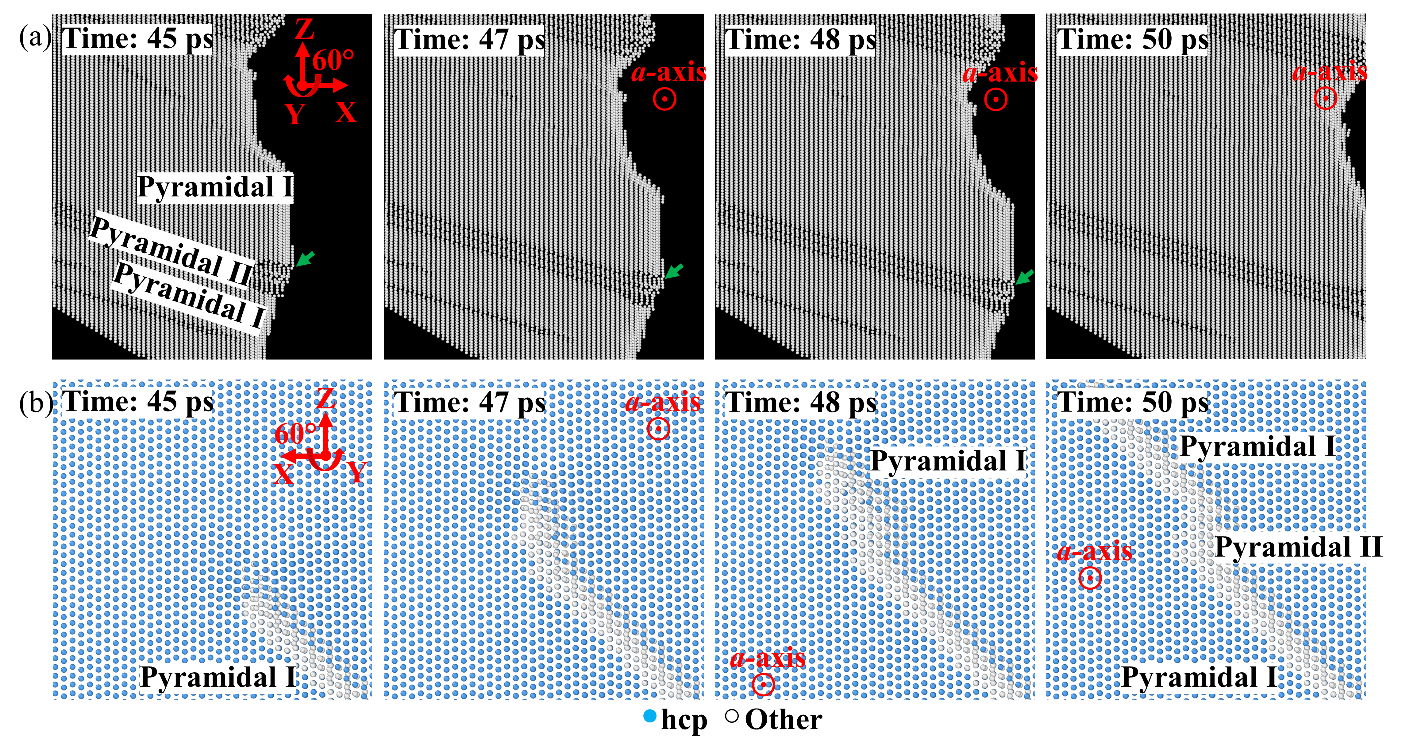


Figure S7. Cross-slip of dislocations from the pyramidal I plane to the pyramidal II plane, shown from an (a) *a*-axis after rotating the simulation box by 60°, and another (b) *a*-axis after rotating the simulation box by -60°. The pyramidal I and pyramidal II stacking faults are marked in both (a) and (b). The green arrows in (a) show where the cross-slip occurs.

**S5. The interfaces that separate the twin from the matrix**

As the twin embryo expands, it reaches the pyramidal I stacking fault below, which limits its growth, and thus the pyramidal I plane becomes one of the interfaces that separates the twin from the matrix. To determine the types of other interfaces, the simulation box is rotated to an orientation at which one of the axes is either parallel or perpendicular to the interface, so that the top view and side view of the interface can be taken. Figure S8 shows that the TBs are usually not coherent, but faceted on other planes, such as the BP/PBs and pyramidal I planes, and this cannot be observed from a 2D projection of twin structures such as those shown in Figure 1 of the main text.


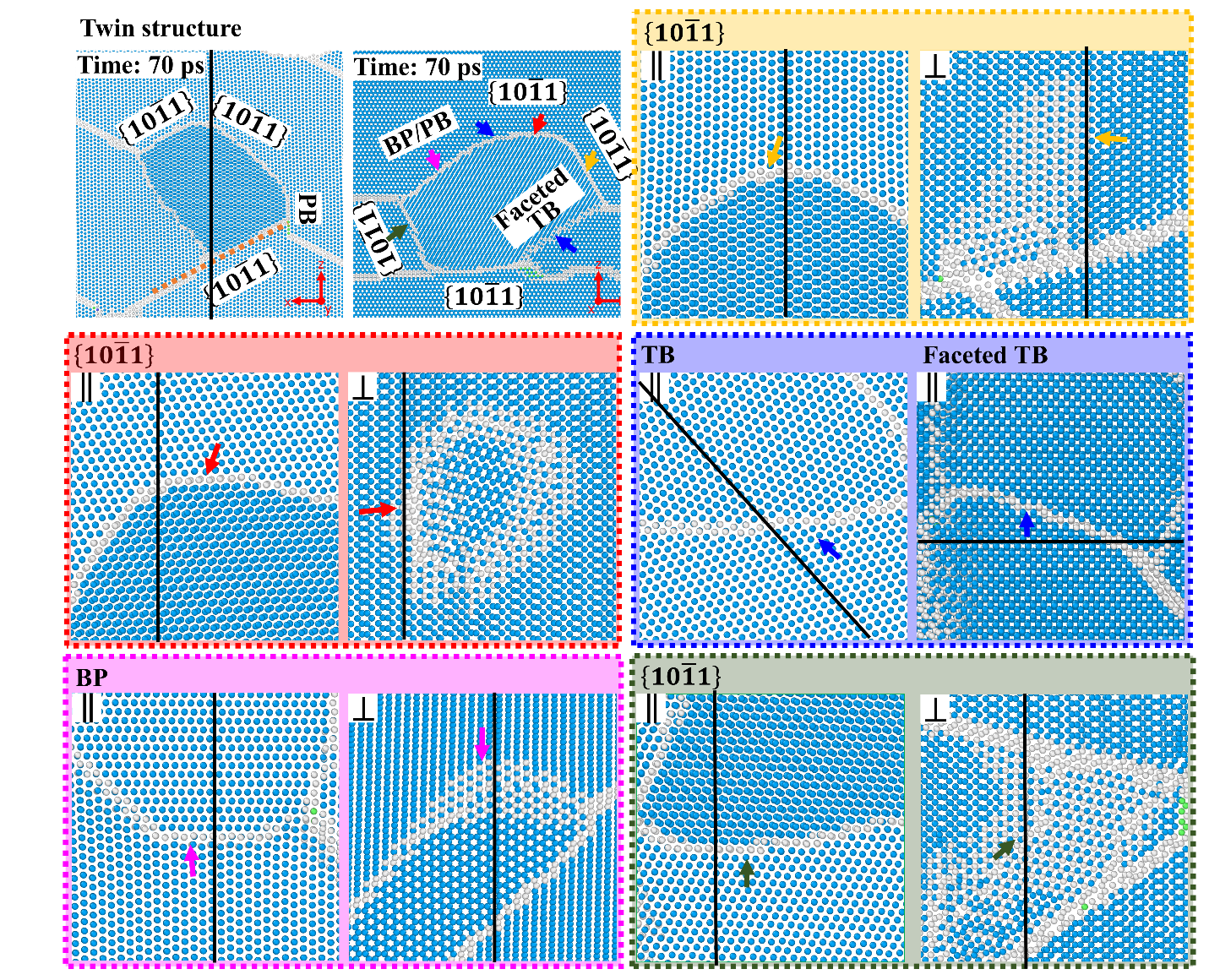


Figure S8. Twin facets that bound the twin embryo at 70 ps. The arrows of different colors mark the various twin facets in the frame at the center of the figure, and the frames outlined by dotted lines of the same color show the side and top views of the corresponding facets, with “║” indicating the side views of facets and “┴” indicating the top views of facets. The solid black line in each frame indicates where the simulation box is cut.

**S6. Other** $\left\{ \boldsymbol{10}\bar{\boldsymbol{1}}\boldsymbol{2} \right\}$ **twins formed during the simulation**

In Figure S9, the dark blue twinned region is small and the misorientation across the boundary that separates the twin and matrix is about 90°. This embryo is not stable and eventually disappears. The $\left\{ 10\bar{1}2 \right\}$ twin embryo that is fully bounded by BP/PB interfaces with the twin lattice 90° from the parent lattice is consistent with the observation of Liu et al. [8] and He et al. [9]. Hu et al. [10] also showed that such twin embryos appear at an early twin growth stage and, as the twin embryo expands, the orientation of basal planes in the twin gradually changes. Therefore, we identify the blue twins as a different $\left\{ 10\bar{1}2 \right\}$ twin variant, highlighted in the hcp unit cell in Figure S18.


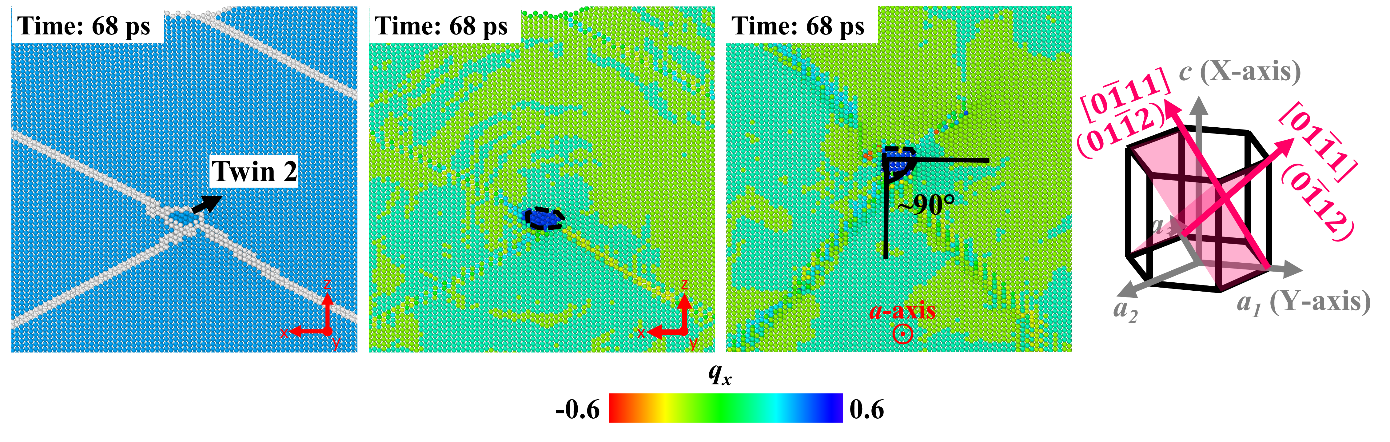


Figure S9. The atomic structure of one $\left\{ 10\bar{1}2 \right\}$ twin activated during the simulation, shown in dark blue. Atoms are colored according to the component *q_x_* of the orientation quaternion. The corresponding twin variant is shown using the unit cells on the right. To determine the twin type that is activated, the simulation box is rotated about the *X*-axis by 60°, so that the *Y*-axis after rotation becomes the *a*-axis shared by both twin and matrix. A 1-nm slab containing the twinned region is then considered.

**S7. The spatial distribution of the normal stress,** $\boldsymbol{\sigma}_{\boldsymbol{XX}}$**, before and after twin nucleation**

The 2D distribution of the atomic stress, $\sigma_{XX}$, before and after twin nucleation is shown in Figure S10, as $\sigma_{XX}$ is the largest stress component among all. Views from the *a*-axis of two orientations are provided. Stress concentrations occur at the surface of the cylindrical void as well as at the dislocation core of the impinging dislocation. At this applied strain level (5.5%), the initial normal stress along the *X*-axis is about 2. 96 GPa, while at ~60 ps (right before twin nucleation), the stress is reduced to ~2.15 GPa due to dislocation activities. The stress at the surface of the cylindrical void or at the dislocation core can reach ~4.3 GPa on average. As the pyramidal I dislocation approaches and intersects with the pyramidal II stacking fault, the stress within the area increases, while initially there is no such high stress concentration at the pyramidal II stacking fault. At ~60.5 ps, the twin already nucleates and the high stress at the intersection is relaxed.

All stress components averaged over selected atoms in the tensile region at the dislocation core are 𝜎_XX_=4.3 GPa, 𝜎_YY_=0.7 GPa, 𝜎_ZZ_=0.8 GPa, 𝜎_XY_=-0.1 GPa, 𝜎_XZ_=-0.5 GPa, 𝜎_YZ_=0.1 GPa. These stress components are then decomposed into the $\left\langle1\bar{1}00 \right\rangle$- and $\left\langle11\bar{2}3 \right\rangle$-directions within the pyramidal II plane, and into the plane normal. The stress tensor after transformation is $\left( \begin{matrix} 0.34 & 0.27 & 0.32 \\ 0.27 & 0.22 & 0.26 \\ 0.32 & 0.26 & 0.31 \end{matrix} \right)$. The three normal components are essential since they help change the size of the pyramidal lattices in the parent, so that the transformation from the pyramidal lattices to the prismatic lattices in the twin can possibly occur.


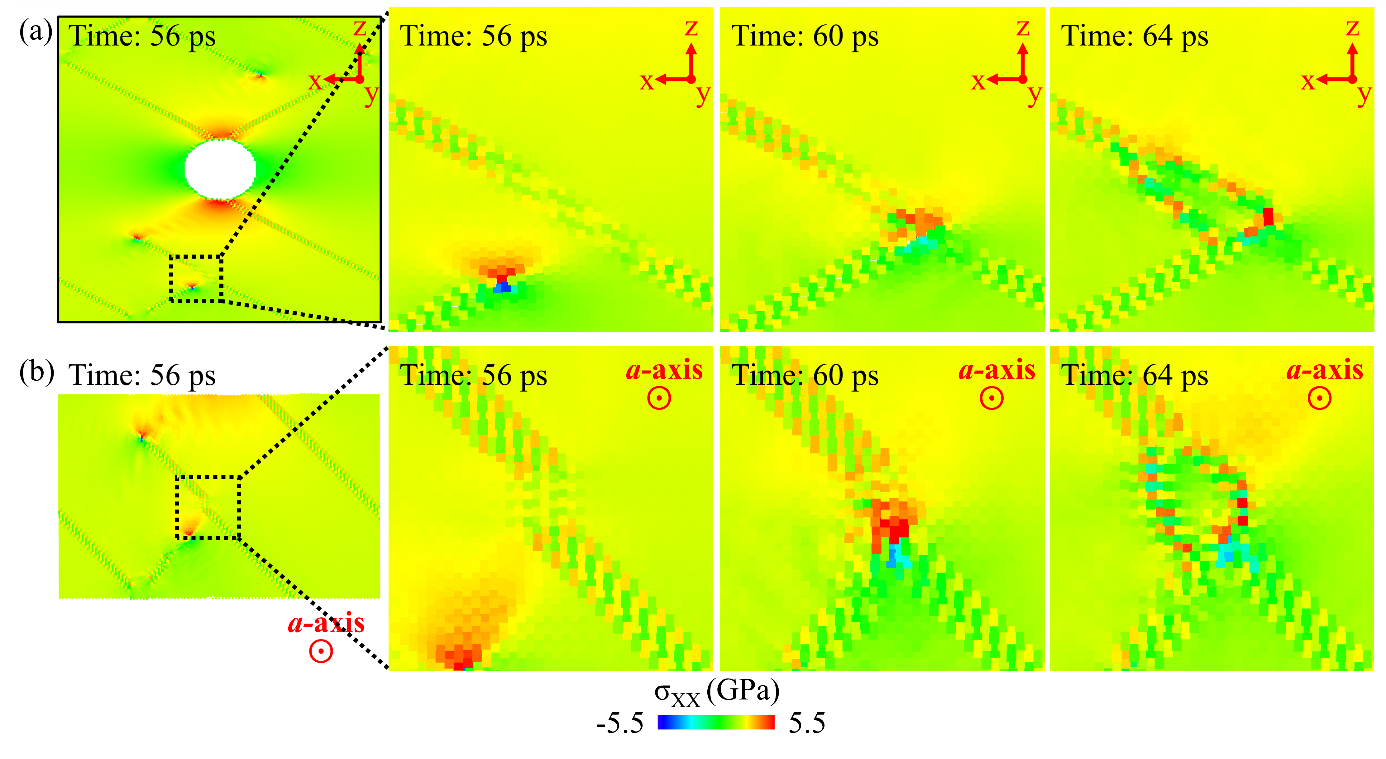


Figure S10. The spatial distribution of the normal stress, $\sigma_{XX}$, viewed from the (a) *Y*-axis and (b) an *a*-axis differently oriented than the *Y*-axis. 1-nm slabs are taken for both views. Dark blue color shows large, negative stresses, while red color shows large, positive stresses.


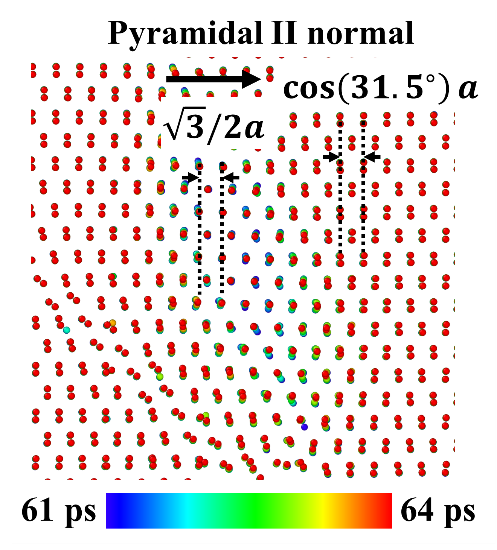


Figure S11. The change of atomic positions during the twin transformation. Atoms are colored according to the simulation time, with dark blue color showing atomic positions at 61 ps and red color showing atomic positions at 63 ps. The size of the pyramidal II and the prismatic lattice along the plane normal is marked.

**S8. The effect of the distribution of atomic velocities**

The same simulations were repeated with different velocity distributions of atoms, and an example of twin nucleation in a different simulation is shown in Figure S12. The twinning process is viewed along the *Z*-axis (Figure S12(a)) and along the *X*-axis (Figure S12(b)) of the simulation box. In the views along the *Z*-axis, only atoms that are in the faulted and twinned regions are shown. In both views, two partial pyramidal I dislocations of the same orientation are generated from the surface of the cylindrical void, and at 7 ps the one on the right starts to cross-slip (see the different arrangement of atoms at the edge of the pyramidal I stacking fault on the right). At 8 ps white atoms appear on a small, tilted plane (Figure S12(b)) with an orientation similar to the one shown in Figure 1. These are the atoms in the faulted region of the pyramidal II stacking fault. The two pyramidal I stacking faults also reach each other and start to merge. At 9 ps the twin embryo is nucleated from the pyramidal II stacking fault. In this example, the twin is formed near the surface of the cylindrical void and not at random locations on the void surface. Figure S12 shows that pyramidal II stacking faults are involved in the twin nucleation process.


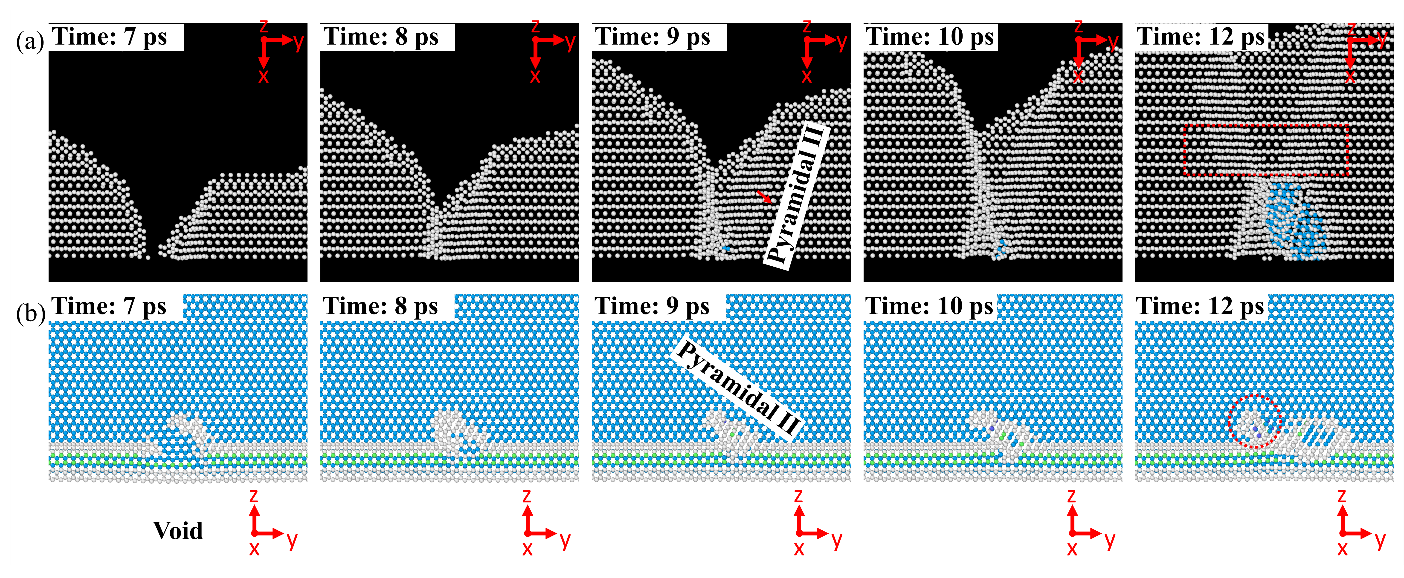


Figure S12. The twin nucleation process viewed from the (a) $\left[ \bar{1}010 \right]$-direction and (b) the *c*-axis in a simulation with different distributions of atomic velocities.

**S9. The effect of temperature**

Low simulation temperatures are used in our work due to the ease of identifying different interfaces and atomic-scale mechanisms. Analogous simulations with cylindrical voids were also performed at 300 K to explore the temperature effect on the proposed twinning mechanism. Two examples of twin nucleation are shown in Figure S13, Video 4 and 5. Higher simulation temperatures lower the energy barrier for dislocation and twin activities; e.g., the onset of pyramidal dislocations at 4% strain is equivalent to a stress of ~1.9 GPa (Figure S13). Figure S14(a) shows a similar twin nucleation assisted by the pyramidal II stacking fault, meaning that the twinning mechanism proposed here is not an unphysical process limited to low temperatures (see also Video 4). At 300 K, the twin nucleation stress is still significantly higher than the values reported from experiments [11,12]. This is unfortunate and due to the size limitations of MD simulations. Experimental works on tensile/compressive testing of Mg nanopillars or micropillars also show an increasing trend in twin nucleation stress with decreasing pillar size. For example, in the work of Della Ventura et al. [13], rectangular pillars of 5 µm × 5 µm × 10 µm were used and deformed at a compressive strain rate of 10^–2^ s^–1^ along the $\left[ 0\bar{1}10 \right]$-direction, and $\left\{ 10\bar{1}2 \right\}$ twins were nucleated at ~100 MPa. In the work of Yu et al. [12], rectangular pillars of 100-200 nm × 150 nm × 2-3 µm were fabricated and deformed at a tensile strain rate of 10^–2^ s^–1^ along the $\left[ 0001 \right]$-direction, and $\left\{ 10\bar{1}2 \right\}$ twins were nucleated from the pillar surface at ~800 MPa.

Figure S14(b) shows one twin that is nucleated near the void surface, and a pyramidal I dislocation is activated preceding twin nucleation (see also Video 5). It is hard to identify whether the pyramidal I dislocation has cross-slipped to the pyramidal II plane due to the fact that twin nucleation occurs not long after the pyramidal I dislocation is emitted from the void surface. In this case, the contribution from pyramidal II stacking fault is uncertain, and twin nucleation could happen via the transformation from the basal lattice to the prismatic lattice.


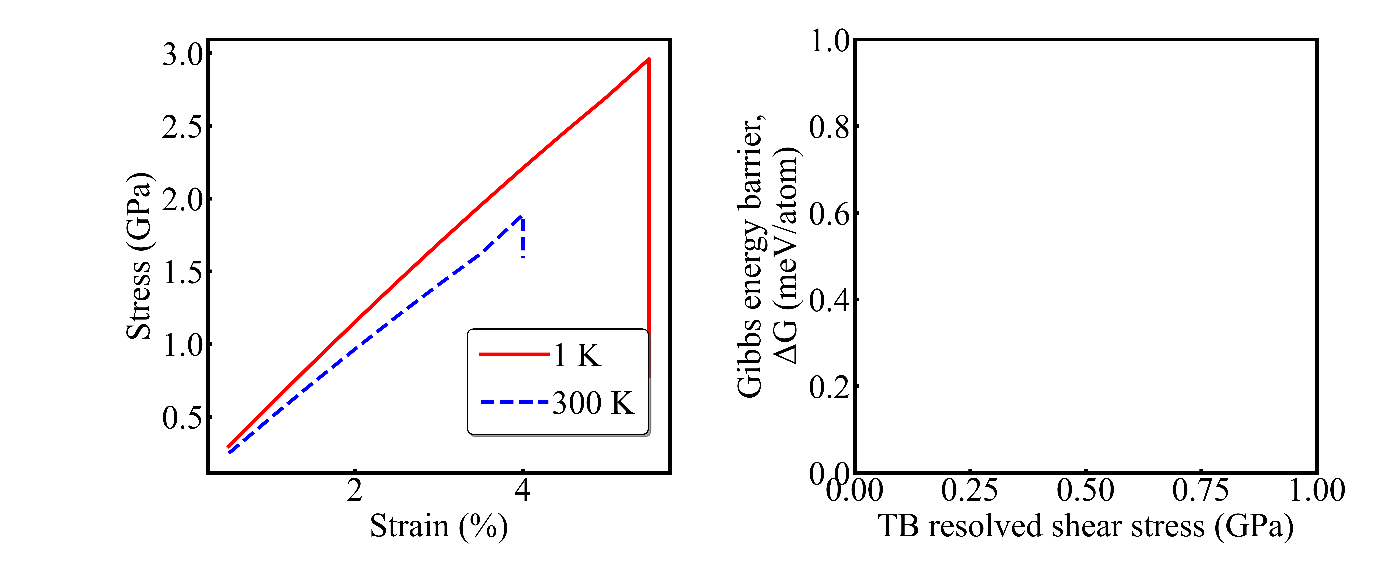


Figure S13. The stress-strain curves for simulations performed at 1 K and at 300 K, in which twin nucleation is activated by applying strain increments along the *X*-axis. The stress drops at 1 K and 300 K are different due to the simulation time used. Since for simulations at 300 K, the onset of twinning occurs around 8-9 ps, being much earlier than simulations at 1 K (~60 ps), the simulations at 300 K were stopped earlier and the stress drops are smaller.


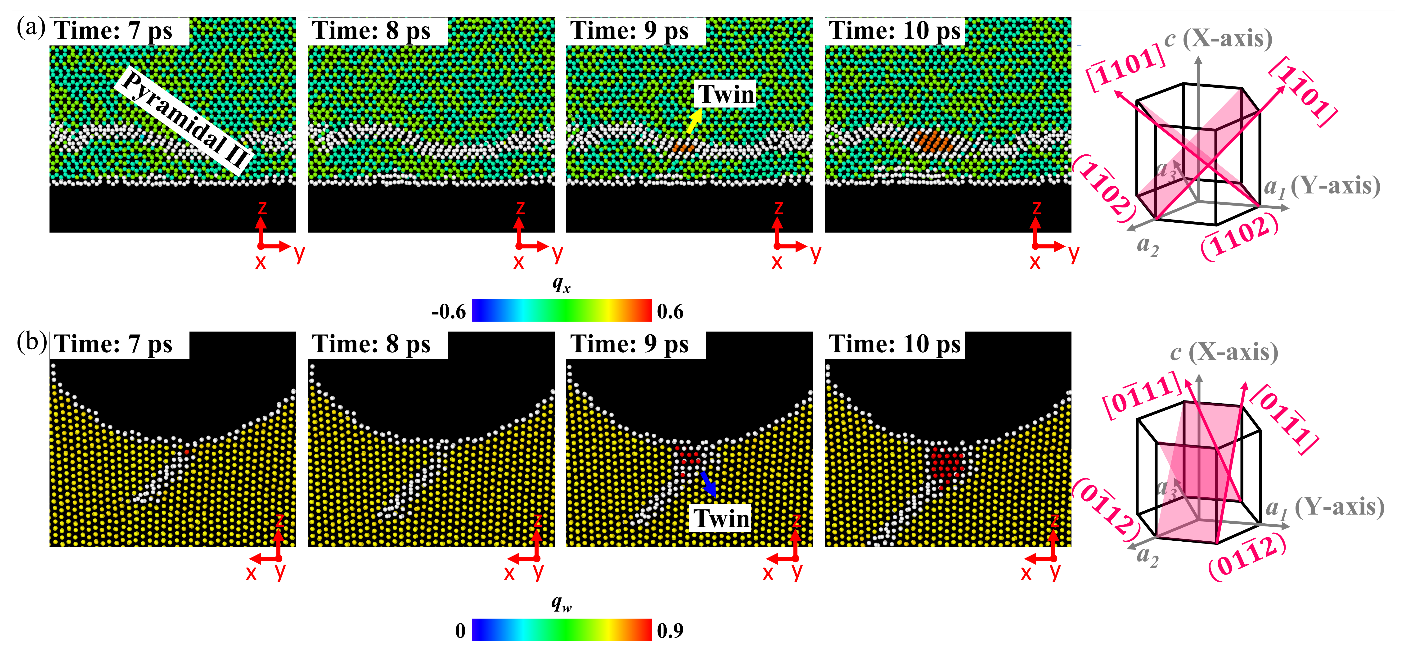


Figure S14. A twin that is nucleated (a) from a pyramidal II stacking fault and (b) near the void surface in simulations at 300 K. The corresponding twin variants for the two twins are shown on the right. In (a) atoms are colored by the lattice orientation component, *q_x_*; in (b) atoms are colored by the lattice orientation component, *q_w_*.

**S10. Twin nucleation from a single pyramidal II stacking fault**

As shown in Figure S15, the simulation box has the same orientation as in Figure S3 of the main text, while the box dimensions are 45.5 × 44.0 × 14.6 nm^3^. To create the same pyramidal II stacking fault as observed in previous simulations, approximately half of the atoms in the box are displaced by the obtained Burgers vector $\frac{2-\sqrt{3}}{3}\left\langle11\bar{2}3 \right\rangle$ along the pyramidal II plane. Some atoms on the boundaries of the simulation box become too close to each other and are therefore deleted. The simulation box is then structurally relaxed at 0 K (molecular statics) with periodic boundary conditions applied to all box dimensions. After ~48,000 steps of energy minimization, the pyramidal II stacking fault with the same atomic arrangement as in previous simulations is observed, and there is a basal stacking fault at the edge of the box, connected to the pyramidal II stacking fault. Such defects are not energetically favorable and eventually transform into stacking faults on a plane with an orientation close to the $\left\{ 11\bar{2}1 \right\}$ plane (see the last frame in Figure S15(a)). According to our calculations, the stacking fault on the $\left\{ 11\bar{2}1 \right\}$ plane has a similar stacking fault energy (~209.58 mJ/m^2^) as the pyramidal II stacking fault (~204.66 mJ/m^2^).

The configuration at the 48,000^th^ minimization step is used for further simulations due to the relatively large area of the stacking fault. Tensile strains are applied to the simulation box by deforming the box uniformly along the *X*-axis. Atoms within ~4 nm from the edge of the simulation box along the *X*-axis are fixed during tensile deformation, since the stacking faults in these regions are no longer on the pyramidal II planes. The rest of the atoms are then relaxed at 0.1 K and at the applied strain. Again, applied strains start from 0.5% and increase gradually, with an increment of 0.5% for every 30 ps. The strain rate is hence 0.5%/30 ps=5/3×10^8^/s (which is, of course, significantly higher than the ones used in experiments). Yet as mentioned earlier, for previous simulations with a cylindrical void, those at 5% strain (the applied strain right before dislocation and twin activities) were prolonged to 2 ns to ensure no defects are activated, meaning that 30 ps is sufficient to allow for the structural relaxation to the energetically most favorable state at the applied strain and temperature. This way of increasing strains gradually guarantees that the configuration is not stuck in a metastable state. A twin is nucleated at 5.5% applied tensile strain, which is equivalent to a stress of ~2.96 GPa. As expected, twin nucleation starts from the stacking fault, and the twin propagates rapidly along the pyramidal II plane, adopting a laminate shape. Thickening of the twin follows, and the major twin facets eventually become faceted TBs, BP/PBs Twist-PyPy1, and Tilt-PyPy1 facets. The energy of the interface between the pyramidal II plane in the matrix and the prismatic plane in the twin ($\left\{ 11\bar{2}2 \right\}_{M}$/$\left\{ 10\bar{1}0 \right\}_{T}$) is high (431.86 mJ/m^2^), compared to other coherent twin facets, which helps explain the quick disappearance of $\left\{ 11\bar{2}2 \right\}_{M}$/$\left\{ 10\bar{1}0 \right\}_{T}$ interfaces during twin growth.


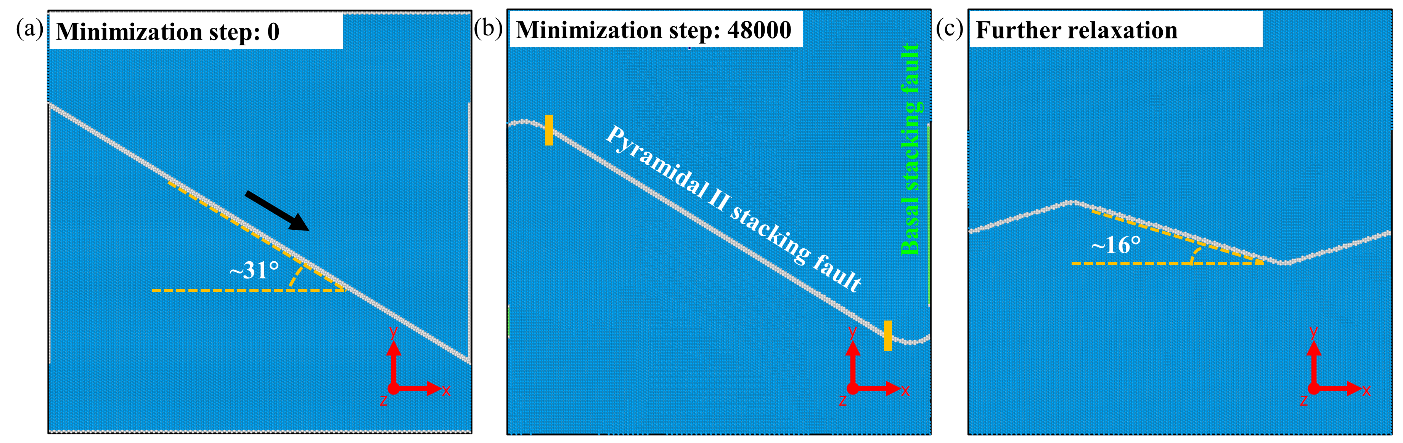


Figure S15. (a) The simulation box, in which approximately half of the atoms are displaced by the Burgers vector $\frac{2-\sqrt{3}}{3}\left\langle11\bar{2}3 \right\rangle$ along the $\left\{ 11\bar{2}2 \right\}$ plane. Some atoms at the edge of the simulation box are too close to each other and are deleted. (b) The atomic structure at the 48,000^th^ minimization step. The pyramidal II stacking fault with the same atomic arrangement as in previous simulations is observed, connected to a basal stacking fault at the edge of the box. This structure was used for further tensile deformation. (c) Stacking faults on the $\left\{ 11\bar{2}1 \right\}$ planes, which are the energetically most favorable structures.

Simulations of twin nucleation from a single pyramidal II stacking fault were repeated using the EAM potential developed by Liu et al. [14] and the MEAM potential of Ahmad et al. [15]. Results are shown in Figure S16. A pyramidal II stacking fault with a large area can be obtained after energy minimization using both potentials, but the structure of the stacking fault is very sensitive to temperature for the case simulated by the EAM potential. The pyramidal II stacking fault almost disappears after keeping it at 0.1 K for 15 ps. The same twin nucleation process is captured by the MEAM potential by applying gradual strain increments. For simulations performed with the EAM potential, twin nucleation can only be achieved by molecular statics simulations at applied tensile strains. This confirms our hypothesis that such a nucleation process is independent of the potentials used, since twin nucleation occurs from the pyramidal II stacking fault irrespective of the potential used in our simulations.


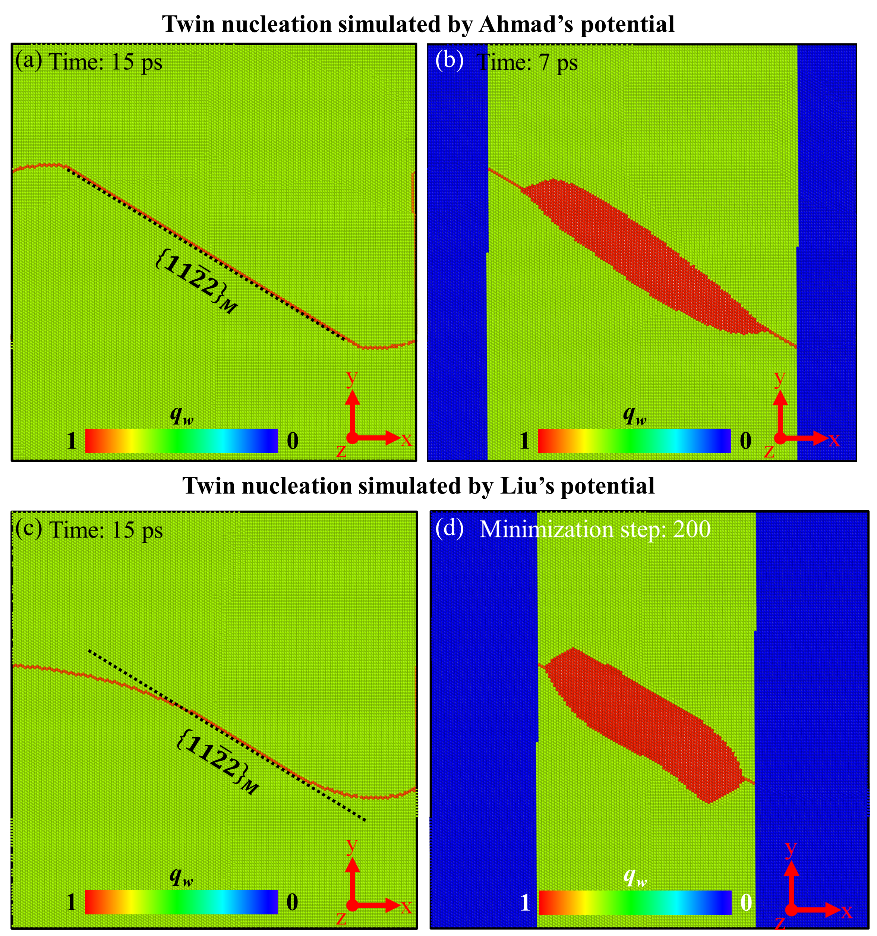


Figure S16. (a) The structure of the pyramidal II stacking fault maintained at 0% strain and 0.1 K for 15 ps, simulated by the Ahmad potential [15]. (b) Twin nucleation from the pyramidal II stacking fault shown in (a), the applied strain is ~7%. (c) The structure of the pyramidal II stacking fault maintained at 0% strain and 0.1 K for 15 ps, simulated by the Liu potential [14]. (d) Twin nucleation from the pyramidal II stacking fault at 5.2% strain and 0 K.

Recent works have introduced the concept of “*unconventional twins*”, which are twins formed from a non-invariant plane under slight distortion [11,16,17]. The work of Della Ventura [34] showed examples of such unconventional twins that develop on $\left\{ 11\bar{2}3 \right\}$ planes or PB/BP interfaces after increasing the strain rate for the compressive testing of Mg micropillars from $\dot{\varepsilon}\leq$10^-2^ s^-1^ to $\dot{\varepsilon}$ >10 s^-1^. Our MD simulations with gradually increasing tensile strains also show a twin nucleation from a non-invariant plane, but such planes cannot be maintained during twin growth due to their high interfacial energies, unlike the experimental observations from [34]. Interestingly, a six-atomic-layer twin (excluding atoms on the interfaces) with the $\left\{ 11\bar{2}2 \right\}_{M}$/$\left\{ 10\bar{1}0 \right\}_{T}$ being the primary boundary that separates twin from the matrix was indeed achieved in our simulations by structurally relaxing the simulation box at ~7% tensile strain. In these simulations, large tensile strains were applied instantaneously in contrast to previous simulations that applied strain increments. Therefore, the strain rate can be considered as infinite, and the twin structure is likely stuck in some metastable state, which could also be the case in [34].


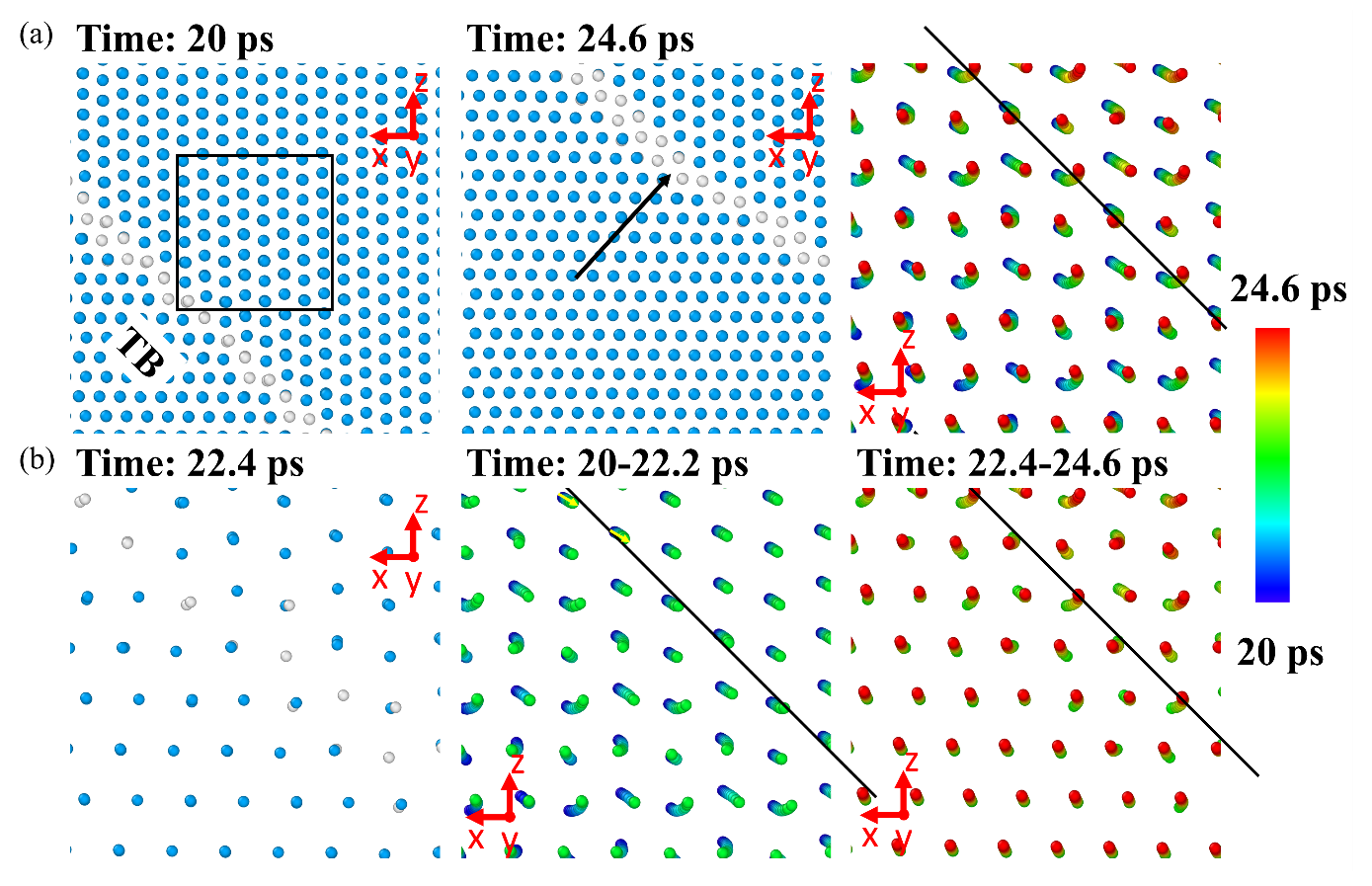


Figure S17. The atomic displacements during TB migration. The black arrow shows the direction of motion of the TB, and the solid black line marks the atoms on one $\left\{ 10\bar{1}2 \right\}$ plane. In (b), the shear and shuffling displacements for atoms on the marked $\left\{ 10\bar{1}2 \right\}$ plane are separated. Atoms undergo only shear displacements before 22.4 ps, and only shuffling displacements afterwards. In the first two frames in (a) and the first frame in (b), hcp atoms are in light blue and atoms on the TB are white. In other frames, atoms are colored according to simulation time, with atoms at 24.6 ps being red and atoms at 20 ps being dark blue.

**S11. Calculation of Gibbs free energy during twin transformation from pyramidal II SF**

The Gibbs free energy is calculated using the following equation,

$G\left( \varepsilon,\sigma,s \right)\equiv U\left( \varepsilon,s \right)-W\left( \varepsilon,\sigma\right)$ (2)

Where $U\left( \varepsilon,s \right)$ is the internal energy and equal to the sum of the potential and kinetic energy, and in our simulations, the kinetic energy is negligible due to low temperature that was used. $W\left( \varepsilon,\sigma\right)$ is the work done by constant external Cauchy stress, *σ*. Below shows the stress-strain curves for the simulations with single pyramidal II stacking fault, and $W\left( \varepsilon,\sigma\right)$ is estimated as 0.5*ε*·*σ*. Figure S18(a) shows the stress-strain curve for simulations with single pyramidal II stacking fault, the stress drop at 5.5% tensile strain corresponds to the twin nucleation. Tensile testing on a simulation box with single pyramidal I stacking fault was also performed for comparison. Even at 15% tensile strain, no transition was observed.


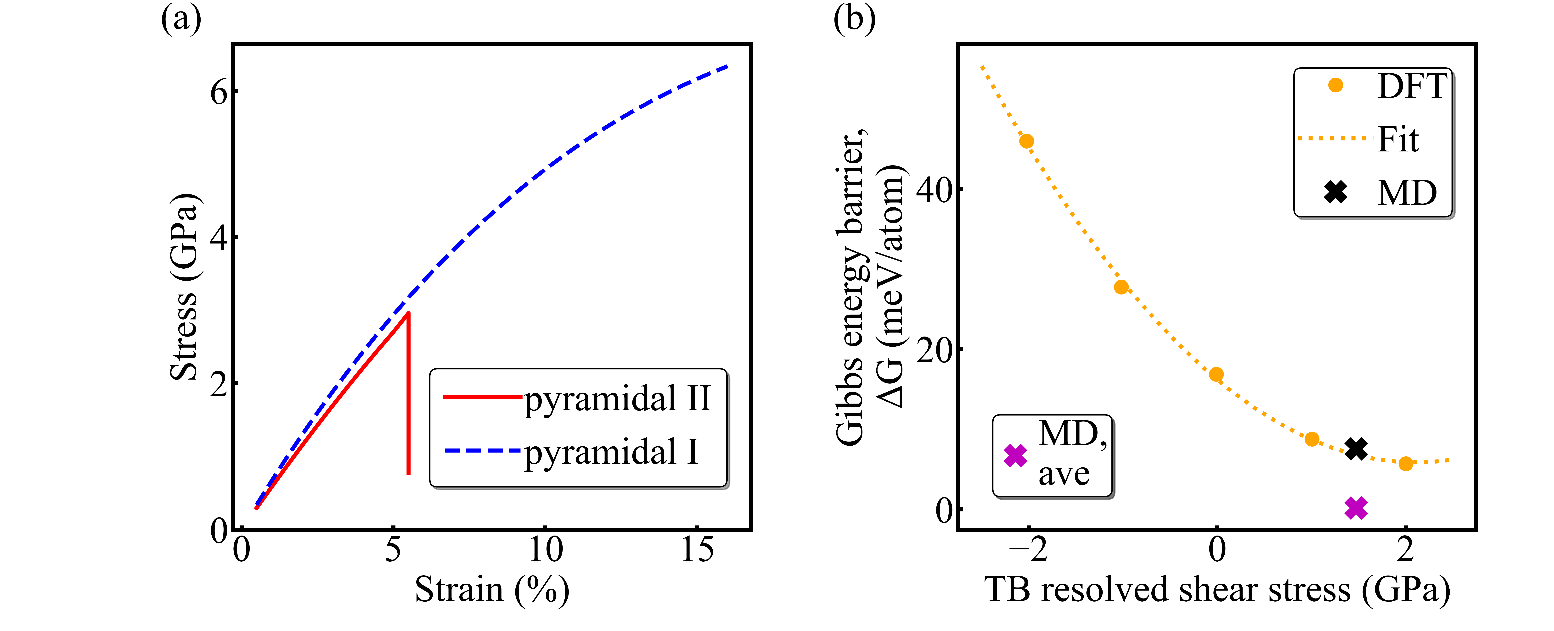


Figure S18. (a) The stress-strain curve for simulations with single pyramidal II stacking fault and simulations with single pyramidal I stacking fault. (b) The variation of Gibbs energy barrier with TB resolved shear stress. The DFT data was fitted using a parabolic fit.

Two ways of estimating the per-atom Gibbs energy barrier were used, the first one is to track the time evolution of Gibbs free energy of the unit and average over eight atoms, and the barrier is near 0. While the second way is to track the change of Gibbs free energy of each atom individually and average over eight atoms, the result is ~7.56 meV, and this data point falls onto the parabolic fit of the Gibbs energy barrier for shuffle-controlled twin nucleation, calculated using the nudged elastic band method with DFT [18] (the fit is only for visualizing the trend).

In [18], Ishii et al. used a four-atom supercell and applied shear stress parallel to the twin plane to activate the transformation. DFT simulations do not output per-atom quantities, the authors track the change of Gibbs energy of the supercell along the deformation path to the energy barrier. Then they averaged the Gibbs free energy barrier over number of atoms and plotted the per-atom value versus the shear stress, which is indeed the twin plane resolved shear stress. In our simulations, a tensile strain along the c-axis is applied and the TB resolved shear stress is calculated using *σ·*cos*ϕ_1_·*cos*ϕ_2_*, where *ϕ_1_* is the angle between and the loading axis and TB normal, and *ϕ_2_* is the angle between the loading axis and the twinning shear direction. The DFT data was fitted by a parabolic fit just for visualization purposes, but there is no report of the relation between Gibbs free energy barrier of twinning and the TB resolved shear stress should be parabolic. The difference between our simulation and the DFT is that, the DFT work studied homogeneous twin nucleation, while the twin nucleates from defects in our case, and thus it is a defect-assisted twin nucleation, which probably also explains the near 0 energy barrier when the same way of averaging energy as in DFT works is used.

Video 1. Slip activity during the deformation of Mg. Shown is the evolution of the atomic structure from 0 ps to 9 ps. hcp atoms are colored in blue, while atoms of other types are white. Partial pyramidal I dislocations are emitted from the surface of the cylindrical void.


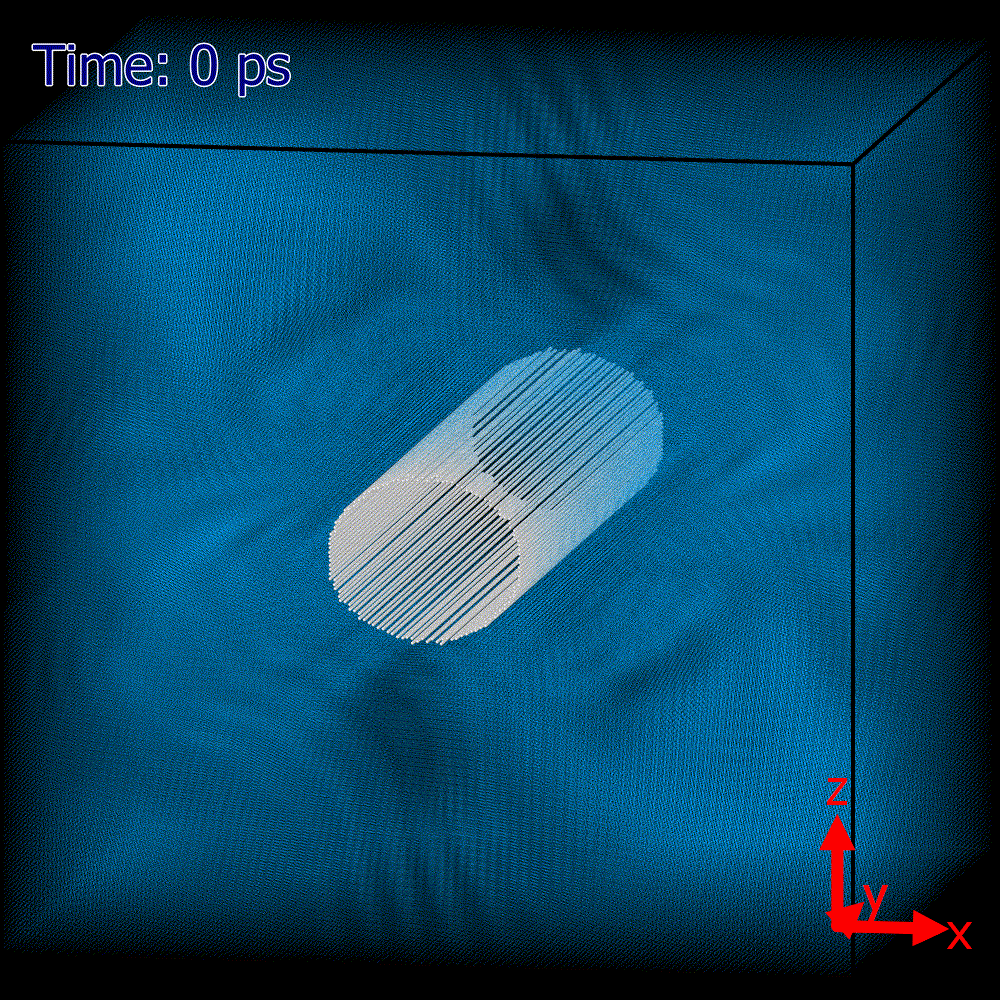


Video 2. Twin nucleation during the deformation of Mg. Shown is the evolution of the atomic structure from 56 ps to 63.5 ps. hcp atoms are colored in blue, while atoms of other types are white. One twin is nucleated at the bottom of the box and is highlighted in red.


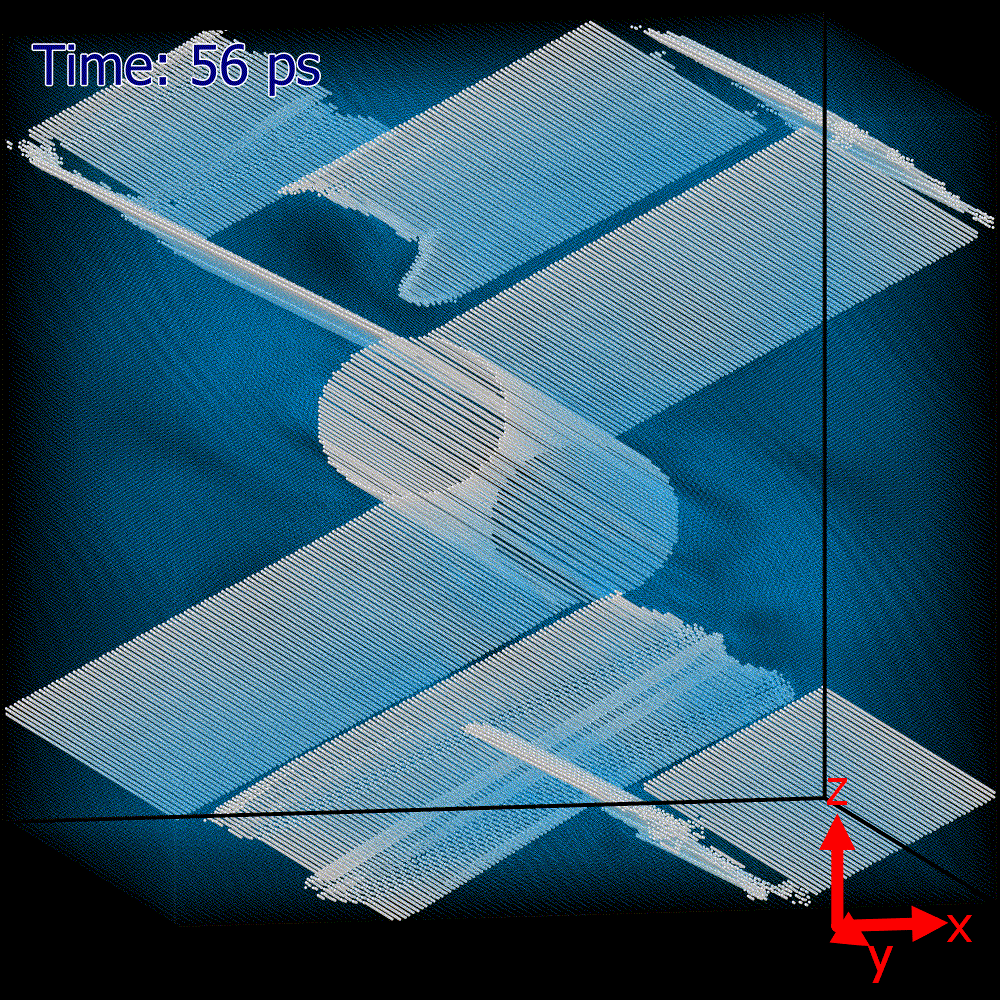


Video 3. Cross-slip of one pyramidal I dislocation to the pyramidal II plane and back to the pyramidal I plane. hcp atoms are shown in blue, while atoms of other types are white. Atomic structures are viewed along a shared *a*-axis after the box is rotated about the *c*-axis (*X*-axis) by -60°. The dislocation moves from the bottom right corner to the top left corner.


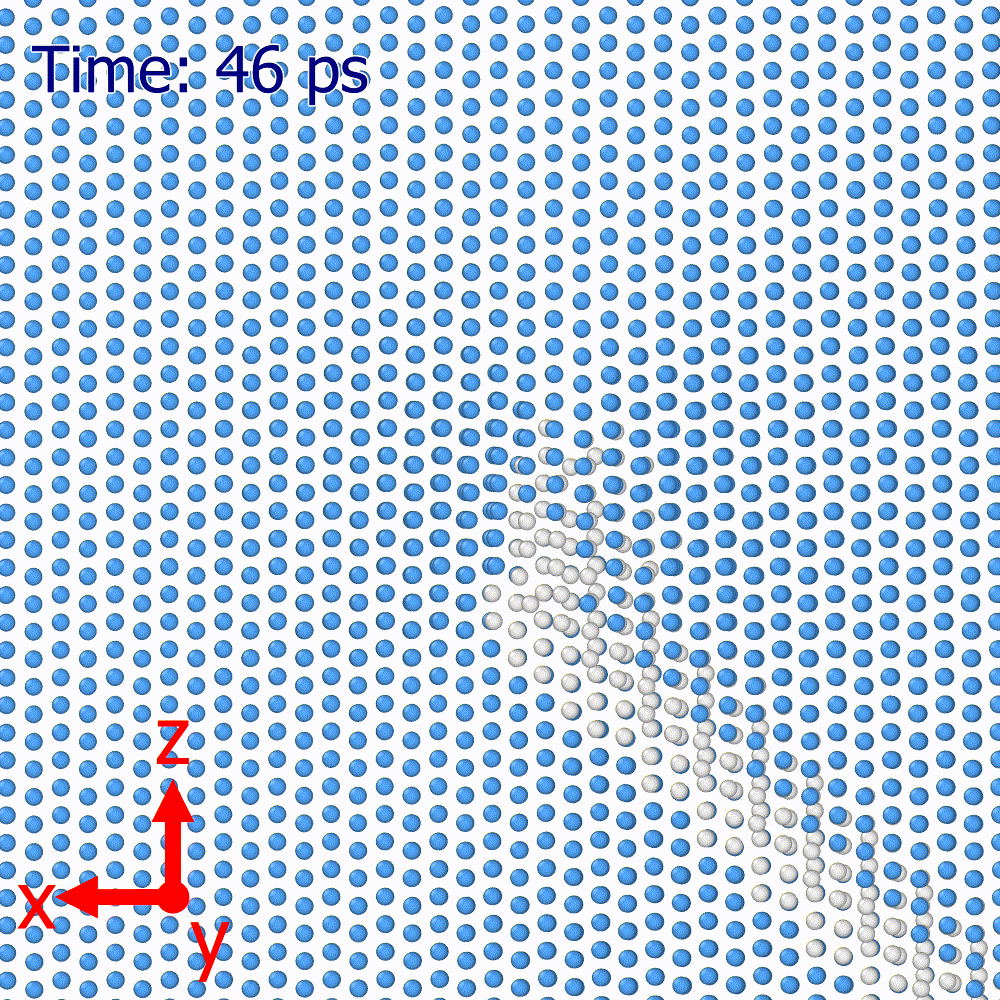


Video 4. One twin nucleation from pyramidal II stacking fault at 300 K. Atomic structures from 4 ps to 18.5 ps are shown, viewed along the *c*-axis (*X*-axis). The twin nucleation occurs at about 14.5 ps. hcp atoms are shown in blue, while atoms of other types are white.


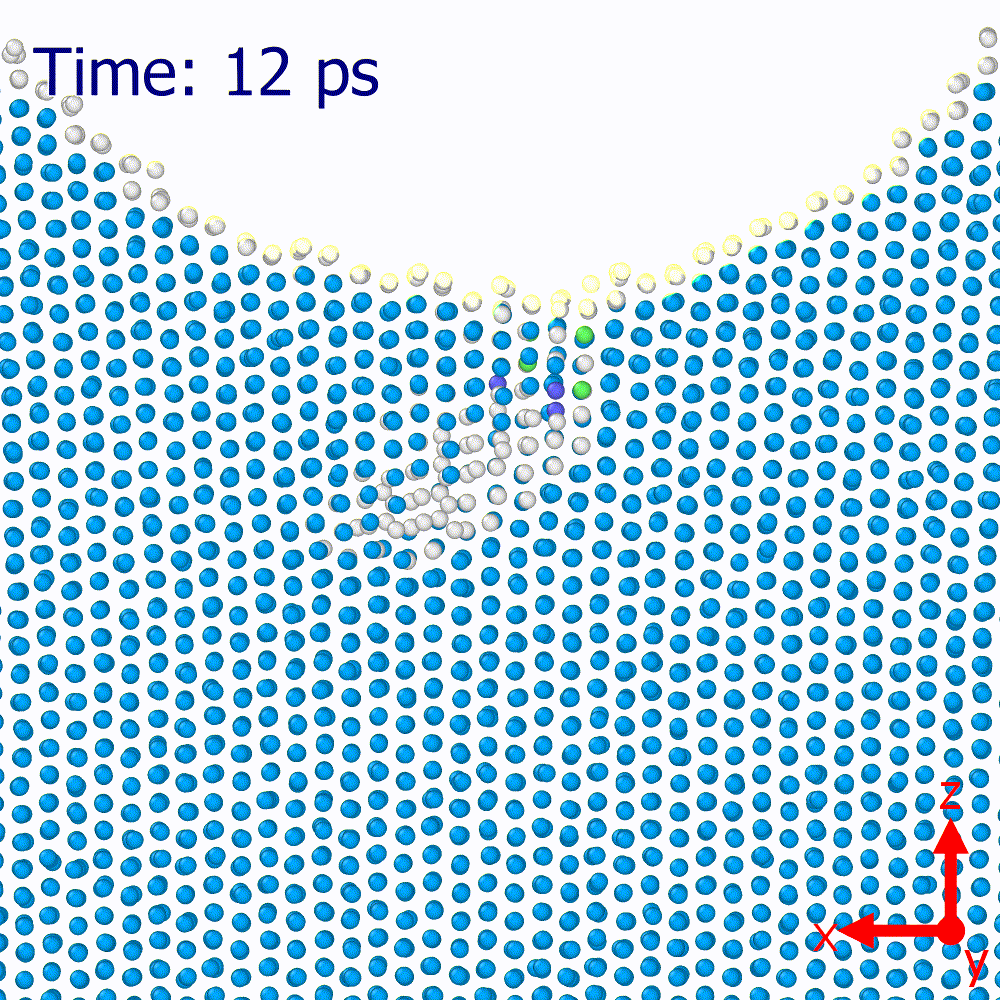


Video 5. Twin nucleation near the void surface at 300 K. Atomic structures from 12 ps to 19.9 ps are shown, viewed along the *a*-axis (*Y*-axis). The basal planes in the matrix (the vertically oriented planes from this perspective) transform into prismatic planes in the twin (the horizontally oriented planes from this perspective). hcp atoms are shown in blue, while atoms of other types are white.

**
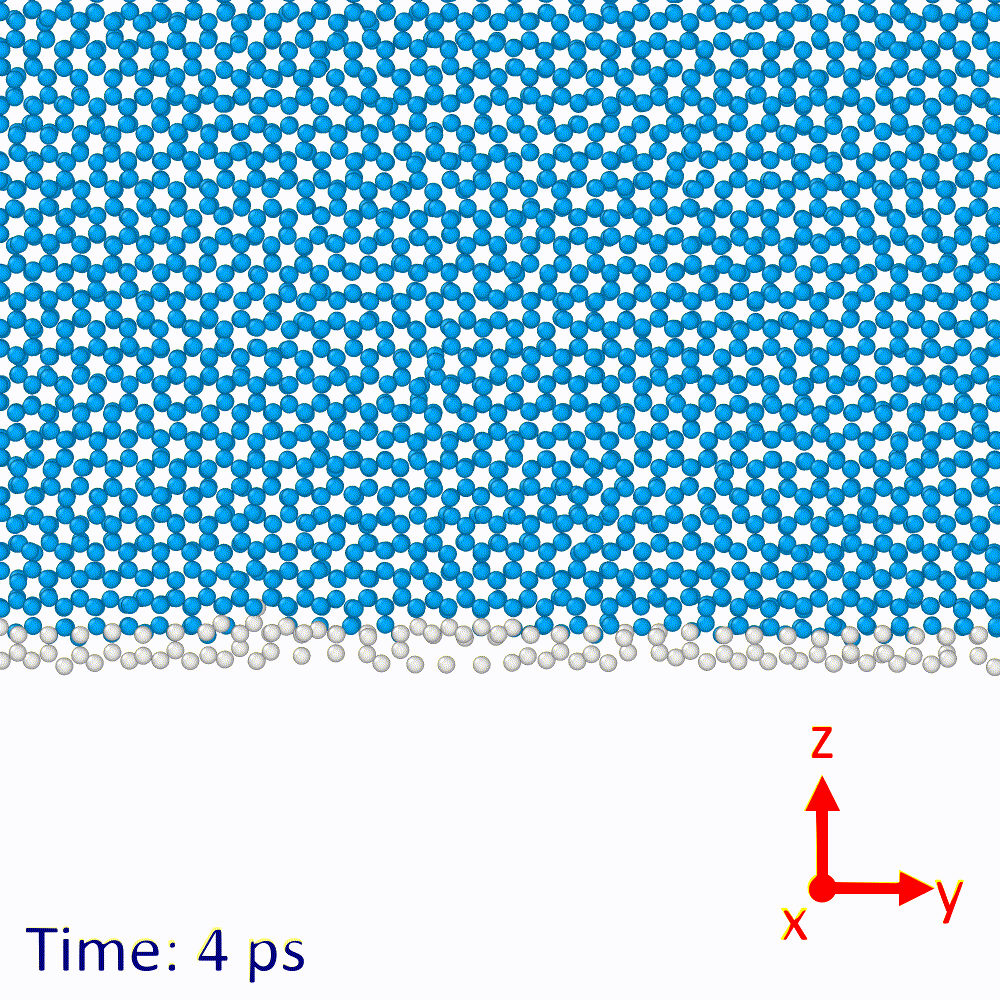
**

**References**

[1] Stukowski A. Visualization and analysis of atomistic simulation data with OVITO–the Open Visualization Tool. Model Simul Mat Sci Eng [Internet]. 2010;18:015012. Available from: https://iopscience.iop.org/article/10.1088/0965-0393/18/1/015012.

[2] Larsen PM, Schmidt S, Schiøtz J. Robust structural identification via polyhedral template matching. Model Simul Mat Sci Eng [Internet]. 2016 [cited 2022 Sep 13];24:055007. Available from: https://iopscience.iop.org/article/10.1088/0965-0393/24/5/055007.

[3] Huang Z, Turlo V, Wang X, et al. Dislocation-induced Y segregation at basal-prismatic interfaces in Mg. Comput Mater Sci. 2021;188.

[4] Gong M, Hirth JP, Liu Y, et al. Interface structures and twinning mechanisms of twins in hexagonal metals. Mater Res Lett [Internet]. 2017;5:449–464. Available from: https://www.tandfonline.com/doi/full/10.1080/21663831.2017.1336496.

[5] Yin B, Wu Z, Curtin WA. Comprehensive first-principles study of stable stacking faults in hcp metals. Acta Mater. 2017;123:223–234.

[6] Nogaret T, Curtin WA, Yasi JA, et al. Atomistic study of edge and screw (c + a) dislocations in magnesium. Acta Mater. 2010;58.

[7] Gong M, Graham J, Taupin V, et al. The effects of stress, temperature and facet structure on growth of {10-12} twins in Mg: A molecular dynamics and phase field study. Acta Mater [Internet]. 2021;208:116603. Available from: https://linkinghub.elsevier.com/retrieve/pii/S1359645420310405.

[8] Liu BY, Wang J, Li B, et al. Twinning-like lattice reorientation without a crystallographic twinning plane. Nat Commun. 2014;5.

[9] He Y, Li B, Wang C, et al. Direct observation of dual-step twinning nucleation in hexagonal close-packed crystals. Nat Commun [Internet]. 2020;11:2483. Available from: http://www.nature.com/articles/s41467-020-16351-0.

[10] Hu Y, Turlo V, Beyerlein IJ, et al. Embracing the Chaos: Alloying Adds Stochasticity to Twin Embryo Growth. Phys Rev Lett. 2020;125:205503.

[11] della Ventura NM, Sharma A, Kalácska S, et al. Evolution of deformation twinning mechanisms in magnesium from low to high strain rates. Mater Des. 2022;217:110646.

[12] Yu Q, Qi L, Chen K, et al. The Nanostructured Origin of Deformation Twinning. Nano Lett [Internet]. 2012;12:887–892. Available from: https://pubs.acs.org/doi/10.1021/nl203937t.

[13] Della Ventura NM, Kalácska S, Casari D, et al. {10-12} twinning mechanism during in situ micro-tensile loading of pure Mg: Role of basal slip and twin-twin interactions. Mater Des. 2021;197:109206.

[14] Liu XY, Adams JB. Grain-boundary segregation in Al-10%Mg alloys at hot working temperatures. Acta Mater. 1998;46.

[15] Ahmad R, Wu Z, Curtin WA. Analysis of double cross-slip of pyramidal I <c+a> screw dislocations and implications for ductility in Mg alloys. Acta Mater. 2020;183.

[16] Cayron C. The concept of axial weak twins. Acta Mater. 2022;236.

[17] Cayron C, Logé R. Evidence of new twinning modes in magnesium questioning the shear paradigm. J Appl Crystallogr. 2018;51.

[18] Ishii A, Li J, Ogata S. Shuffling-controlled versus strain-controlled deformation twinning: The case for HCP Mg twin nucleation. Int J Plast. 2016;82.
